# Elucidating the phytochemical profile of Sophorae Flavescentis Radix-Astragali Radix herb pair: an integrated LC-QTOF-MS/MS, pharmacological activity, and network pharmacology study on anti-hepatocellular carcinoma effects

**DOI:** 10.3389/fchem.2025.1687098

**Published:** 2025-11-07

**Authors:** Yidi Yin, Jiameng Qu, Lanzhuo Qu, Zhiyuan Li, Huarong Xu, Qing Li

**Affiliations:** 1 National and Local Joint Engineering Laboratory for Key Technology of Chinese Material Medica Quality Control, School of Pharmacy, Shenyang Pharmaceutical University, Shenyang, China; 2 SCIEX China, Shanghai, China

**Keywords:** Sophorae Flavescentis Radix-Astragali Radix, hepatocellular carcinoma, phytochemical profiling, LC-QTOF-MS, network pharmacology

## Abstract

**Background:**

Hepatocellular carcinoma (HCC) remains refractory because recurrence, drug resistance and systemic toxicity limit current therapeutics. The traditional herb pair Sophorae Flavescentis Radix-Astragali Radix (SF-AR) is reputed to counter liver disorders, but its anti-HCC potential and chemical basis have not been delineated.

**Methods:**

Anti-tumor activity was assessed in HepG2 cells and an H22 allograft mouse model. Constituents were characterized by high-performance liquid-chromatography–quadrupole time-of-flight mass spectrometry, and bioavailable prototypes were traced in rat plasma and organs. Absorbed molecules were linked to HCC-related targets through network pharmacology, and molecular docking examined their interactions to suggest potential target engagement.

**Results:**

SF-AR suppressed HepG2 proliferation, abolished colony formation and induced dose-dependent apoptosis without harming L02 normal hepatocytes. Oral administration reduced H22 tumor burden in a dose-responsive manner without overt toxicity under the study conditions. Ninety-five constituents were delineated, encompassing 37 flavonoids, 23 alkaloids, 12 terpenoids, and organic, amino and nucleic acids; class-specific collision-induced dissociation pathways were summarized, including RDA cleavages for isoflavonoids and diagnostic ions for matrine-type alkaloids. Following oral administration, twenty-two prototype constituents were detected in rat plasma and were preferentially distributed to liver, kidney and spleen, confirming systemic exposure. Network pharmacology associated the absorbed constituents with potential HCC-related targets, and inflammation- and survival-related pathways were implicated as potential targets; favorable binding of representative constituents to these proteins was supported by molecular docking.

**Conclusion:**

Integrated pharmacological, chemical and computational evidence links the measurable anti-HCC efficacy of SF-AR to a bioavailable, multi-class phytochemical ensemble that converges on inflammation-driven survival pathways. SF-AR therefore emerges as a multi-target, low-toxicity therapeutic candidate that warrants further preclinical development for hepatocellular carcinoma.

## 1 Introduction

Hepatocellular carcinoma (HCC) is the most prevalent primary liver malignancy and a leading cause of cancer-related mortality globally. Despite diagnostic and therapeutic advancements, the prognosis for patients with HCC, particularly those diagnosed at advanced stages, remains poor (Rumgay et al., 2022). Current treatment modalities include surgical resection, liver transplantation, systemic chemotherapy, and targeted agents such as tyrosine kinase inhibitors and immune checkpoint inhibitors. However, the clinical efficacy of these interventions is frequently constrained by disease aggressiveness, high recurrence rates, and notable side effects, highlighting an urgent need for more effective and less toxic therapeutic strategies (Hartke et al., 2017). Against this backdrop, traditional Chinese medicine (TCM) therapies, known for their multi-constituents, multi-therapeutic targets, and fewer side effects (Liu et al., 2019; Yang Z. et al., 2021), are utilizing natural herbs to target multiple biological pathways based on holistic principles (Qi et al., 2010; Wong et al., 2001). TCM has emerged as a complementary or alternative approach for managing various cancers, including HCC (Chen C. et al., 2021; Lam et al., 2015; Shao et al., 2024; Xi and Minuk, 2018). Within the TCM armamentarium, Sophorae Flavescentis Radix (SF) and Astragali Radix (AR) are well-established herbal medicines recognized for their anti-inflammatory, immunomodulatory, and anti-cancer properties. Both herbs have individually demonstrated significant anti-cancer potential in *vitro* and *in vivo* studies.

Sophorae Flavescentis Radix (SF), derived from the dried root of *Sophora flavescens* Ait. and known in Chinese as Ku Shen, has a long history in TCM, where it is traditionally used for indications described as heat-clearing, dampness-expelling, and parasite-killing. It is a component of various herbal formulations (He et al., 2015; Sun et al., 2022). Phytochemical analyses have identified key bioactive constituents in SF, including alkaloids such as matrine and oxymatrine (Ying et al., 2015), and flavonoids such as kurarinone (Dong et al., 2021; Yu et al., 2013). Matrine, for instance, demonstrates anti-HCC activity through multiple mechanisms: it induces apoptosis, inhibits angiogenesis, curtails cell invasion and migration, and enhances the sensitivity of HCC cells to chemotherapy (Du et al., 2020). Oxymatrine exhibits comparable anti-HCC effects and further impedes HCC progression by modulating the AKT and EGFR signaling pathways. Kurarinone, a prenylated flavonoid, has been shown to inhibit HCC cell proliferation and induce apoptosis, potentially via suppression of NF-κB signaling (Han et al., 2007). AR, or *Huang Qi* in Chinese, is sourced from the dried roots of *Astragalus membranaceus* (Fisch.) Bge. var. *mongholicus* (Bge.) Hsiao or *A. membranaceus* (Fisch.) Bge. AR is recognized for its immune-enhancing capabilities, primarily attributed to B-cell proliferation stimulus and cytokine production modulation (Chen et al., 2020; Xu et al., 2018; Zhao et al., 2011). Furthermore, AR influences key signaling pathways, notably MAPK (Xu et al., 2019) and NF-κB (Lee and Jeon, 2005). The herb is abundant in flavonoids and saponins, with calycosin and astragaloside IV being prominent examples known for their anti-inflammatory and anti-proliferative activities (Chen T. et al., 2021; Lu et al., 2024; Yang et al., 2013).

A distinctive feature in TCM is the use of herb pairs - two herbs used together to enhance therapeutic effects while reducing potential toxicity. Balanced pairing is essential not only for efficacy but also for safety in TCM applications (Wang et al., 2012). It is hypothesized that SF and AR, when used in combination, may enhance each other’s therapeutic properties, thereby improving overall anti-cancer efficacy. The anti-HCC effect of the SF-AR herb pair is well documented in ancient literature and has been corroborated by our preliminary experiments (Yang et al., 2024). Furthermore, the primary active constituents of SF and AR have been developed into clinical preparations. *Kangai Injection*, consisting of AR, panax ginseng and matrine, as well as *Compound Kushen Injection* have been approved by the China NMPA for the treatment of cancer and chronic hepatitis, playing a supportive role in cancer treatment (Gao et al., 2018; Song et al., 2023; Yang M. et al., 2021).

Although individual studies have investigated the anti-cancer effects of SF and AR, research specifically addressing their combined action as the SF-AR herb pair in HCC remains limited. Consequently, the precise anti-HCC effects of SF-AR and its underlying molecular mechanisms require further elucidation. Moreover, despite indications of therapeutic potential, the comprehensive chemical basis of the SF-AR herb pair is not yet fully understood. Existing research has predominantly focused on select individual constituents, thereby lacking a holistic analysis of the pair’s complete chemical profile. This analytical challenge is compounded by the prevalence of isomers within both herbs, underscoring the necessity for advanced qualitative methodologies. In this regard, high-performance liquid chromatography (HPLC) coupled with high-resolution mass spectrometry (HRMS) offers a robust platform for the detailed characterization of complex phytochemical mixtures inherent in herbal medicine (Stark et al., 2019; Wang et al., 2021).

Chronic, dysregulated inflammation is widely recognized as a pivotal driver of tumor initiation and progression, critically shaping the tumor microenvironment via sustained cytokine release, oxidative stress, and activation of pro-survival signaling pathways (Wang et al., 2024). Furthermore, aberrant inflammatory pathways not only contribute to intrinsic malignancy but also promote resistance to conventional anticancer therapeutics, rendering them compelling targets for multi-component interventions. Consequently, targeting inflammation-related pathways has emerged as a viable therapeutic strategy for HCC. Based on these premises and the known properties of SF and AR, we hypothesized that the SF-AR herb pair exerts its anti-HCC effects primarily through the modulation of critical inflammation-linked signaling cascades, such as the PI3K-Akt/NF-κB pathway. While existing evidence indicates that SF and AR, or their individual active constituents, can influence inflammation-related pathways in HCC, the precise downstream pathways affected by the combined SF-AR formulation and its specific role in regulating tumor growth remain to be fully elucidated. To address such complexities, network pharmacology offers a powerful approach, enabling the construction of comprehensive “compound-target-disease” interaction maps from online databases. This methodology facilitates the elucidation of molecular interactions between the complex chemical systems of TCM and relevant biological networks, thereby providing novel insights into their mechanisms of action.

**FIGURE 1:**
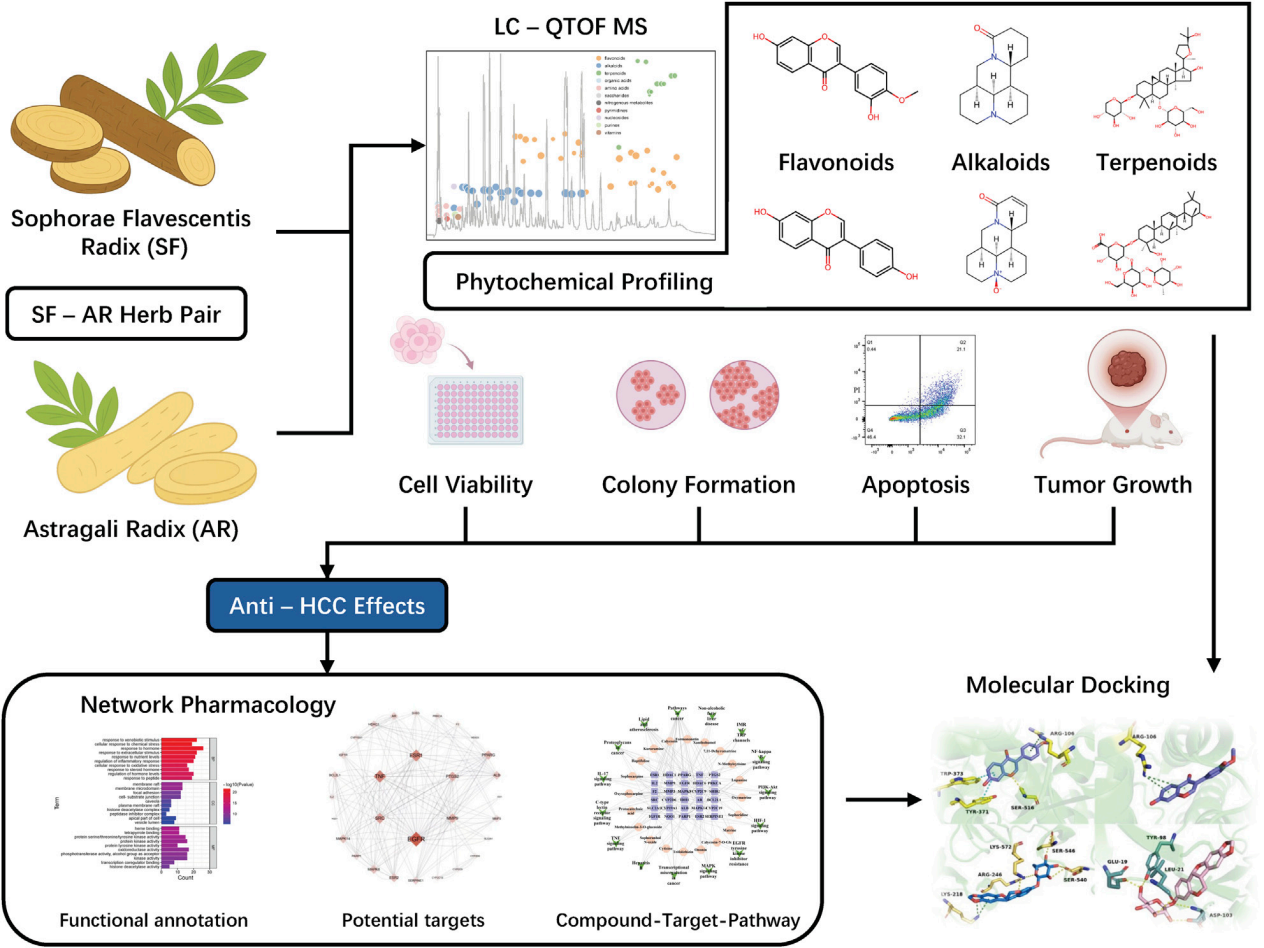
Integrated workflow for the SF-AR herb pair study against HCC.

To address the challenges, this study was designed to systematically evaluate the anti-HCC efficacy of the SF-AR herb pair using both *in vitro* and *in vivo* approaches, focusing on cell proliferation, apoptosis induction, and tumor growth inhibition. A critical component of this investigation involved the comprehensive characterization of the chemical constituents within the SF-AR herb pair using liquid chromatography coupled with quadrupole time-of-flight mass spectrometry (LC-QTOF-MS). Furthermore, the *in vivo* absorption profile of SF-AR’s constituents was determined and integrated with network pharmacology analyses to enhance the predictive reliability of its anti-HCC mechanisms. This absorption-based approach is critical for ensuring that the subsequent mechanistic predictions are grounded in biologically relevant compounds that achieve systemic exposure. This multifaceted strategy, encompassing compound-target prediction, gene function annotation, and pathway enrichment analysis, aimed to uncover key molecular targets and signaling pathways mediating SF-AR’s therapeutic effects. Molecular docking studies were also used to further investigate interactions between potential active constituents and their predicted targets. By synergizing traditional herbal knowledge with advanced analytical and computational methodologies, this research seeks to provide a deeper understanding of the SF-AR herb pair’s bioactive components and their mechanisms of action. Ultimately, the findings are anticipated to offer novel insights into integrating TCM with modern therapeutic paradigms, contributing to the development of more effective and less toxic strategies for HCC, a disease with an urgent need for improved treatment options. The overall study workflow is shown in Figure 1.

## 2 Materials and methods

### 2.1 Preparation of herbal extracts

Extracts of the SF-AR herb pair, SF alone, and AR alone were prepared following the *Practice for the Administration of Decoction Departments for TCM in Medical Institutions*, issued by the National Administration of Traditional Chinese Medicine of China. For the SF-AR herb pair, accurately weighed amounts of SF and AR were combined in a 3:1 (*w/w*) ratio and thoroughly mixed. The mixed crude drugs were soaked in an 8-fold volume (*v/w*) of distilled water relative to the total herb weight for 40 min, then refluxed for 1 h. This extraction was repeated once with a 6-fold volume (*v/w*) of distilled water for an additional 1 h. The filtrates from both extractions were combined and concentrated *in vacuo* at 65 °C to achieve a final concentration of 1.0 g/mL, based on the original crude drug weight. For comparative purposes, individual extracts of SF and AR were prepared using the identical procedure, each yielding a final concentration of 1.0 g/mL. All concentrated extracts were aliquoted and stored at 4 °C until required for analysis or administration. These extracts were subsequently analyzed by LC-QTOF-MS or utilized for administration. The information of the chemicals and reagents as well as the source of the herbs are detailed in Supplementary Material and Supplementary Table S1.

### 2.2 Anti-HCC efficacy assessment using cell models

#### 2.2.1 Cell culture

The human hepatocellular carcinoma cell line HepG2 was obtained from the Tumor Cell Bank of the Chinese Academy of Sciences (Shanghai, China), and the human normal hepatic cell line L02 was procured from Meilunbio Co., Ltd. (Dalian, China). HepG2 cells were maintained in MEM medium (Hyclone, UT, United States) supplemented with 10% fetal bovine serum (FBS; Gibco, NY, United States) and 1% penicillin-streptomycin solution (Hyclone, UT, United States). L02 cells were cultured in RPMI-1640 medium (Hyclone, UT, United States) containing 20% FBS and 1% penicillin-streptomycin. Both cell lines were incubated at 37 °C in a humidified atmosphere of 5% CO_2_ (Model 3111 CO_2_ incubator, Thermo, CA, United States).

#### 2.2.2 Cell viability assay

Cells were seeded into 96-well plates (Thermo, CA, United States) at 5,000 cells/well and, after adherence, were treated with SF-AR herb pair extract at final concentrations of 10.0, 20.0, or 30.0 mg/mL for 24, 48, or 72 h. Post-incubation, cell viability was quantified using a methyl thiazolyl tetrazolium (MTT) assay according to previously described (Qu et al., 2022). Absorbance was measured using an Infinite 200 Pro microplate reader (Tecan, Salzburg, Austria).

#### 2.2.3 Colony formation assay

The HepG2 cells were seeded in 6-well plates (Thermo, CA, United States) at 1,000 cells/well and allowed to attach for 24 h. Subsequently, cells were exposed to SF-AR extract (10.0, 20.0, or 30.0 mg/mL) for 48 h. The treatment medium was then replaced with fresh complete medium, and cells were cultured for an additional 14 days to allow colony development. Colonies were subsequently fixed with methanol, stained with 1% crystal violet solution (Solarbio, Beijing, China), and manually counted under an optical microscope (Motic, Xiamen, China).

#### 2.2.4 Apoptosis assay

HepG2 cells were seeded in 6-well plates (Thermo, CA, United States) at a density of 4 × 10^5^ cells/well and cultured for 24 h. Cells were then treated with SF-AR extract (10.0, 20.0, or 30.0 mg/mL) for 48 h. Following treatment, both the culture medium and drug solution were aspirated, and cells were washed with PBS. Cells were harvested by digestion with 0.25% trypsin (EDTA-free), and the enzymatic reaction was neutralized with complete culture medium. Collected cells were centrifuged at 2000 rpm for 5 min at 4 °C, and the supernatant was discarded. After two washes with ice-cold PBS, cells were gently resuspended in an appropriate volume of binding buffer. A 500 μL aliquot of the cell suspension was incubated with 5 μL of Annexin V-FITC and 10 μL of propidium iodide solution in the dark at room temperature for 5 min, followed by analysis using a FACSVerse™ flow cytometer (BD Biosciences, NJ, United States).

### 2.3 Anti-HCC efficacy assessment using animal models

#### 2.3.1 Animals and experimental grouping

Forty-two male Institute of Cancer Research (ICR) mice (20 ± 2 g body weight) were procured from Liaoning Changsheng Biotechnology Co., Ltd. (Benxi, China, certificate NO. SCXK (Liao) 2020–0001). The mice were housed individually in a specific pathogen-free (SPF) animal facility under controlled conditions (25 °C ± 2 °C, 40%–60% humidity, 12-h light/dark cycle) with *ad libitum* access to standard chow and water. The murine hepatocellular carcinoma H22 cell line was obtained from Hunan Fenghui Biotechnology Co., Ltd. (Hunan, China).

All animal experiments were conducted in strict accordance with the guidelines established by the Animal Care and Use Committee of Shenyang Pharmaceutical University and followed previously reported protocols (Chang et al., 2018). Briefly, a mouse tumor allograft model was established by subcutaneous injecting of 3 × 10^7^ H22 cells in 100 µL normal saline into the right flank of mice on day 1. After 24 h, the inoculated mice were randomly divided into seven groups (n = 6): a model group treated with distilled water (0.1 mL/10 g/d); a positive control group receiving 5-fluorouracil (5-FU) at 10 mg/kg, intraperitoneally every 2 days starting on day 5; SF alone group (SF, 3.75 g/kg/d); AR alone group (AR, 1.25 g/kg/d); and three groups receiving the SF-AR herb pair at different concentrations (low dose, SF-AR_L, 2.5 g/kg/d; medium dose, SF-AR_M, 5.0 g/kg/d; high dose, SF-AR_H, 10.0 g/kg/d). The treatment lasted for 14 days. Mice were weighed and then euthanized 12 h after the final dose, by cervical dislocation performed by trained personnel. Death was confirmed by cessation of heartbeat and respiration and loss of corneal and pedal reflexes. The tumor tissues were collected for further analysis.

#### 2.3.2 Tumor inhibition rate determination

The tumor inhibition rate was calculated using the formula:

$$Inhibition\;rate=\left(1-\frac{W1}{W2}\right)\times 100\%$$

Where: W1: average tumor weight of the treatment groups; W2: average tumor weight of the model groups.

#### 2.3.3 Histopathological evaluation

Tumor tissues were fixed in 4% paraformaldehyde and embedded in paraffin. Paraffin-embedded tumor samples were sectioned into 3–5 μm slices using an ultramicrotome and stained with hematoxylin and eosin (H&E). Pathological changes were assessed under an inverted fluorescence microscope (Nikon, Japan).

### 2.4 Investigation of absorbed and distributed constituents of SF-AR herb pair in rat

#### 2.4.1 Animals and experimental grouping

Twenty-four male Sprague-Dawley rats (200 ± 20 g) were obtained from Liaoning Changsheng Biotechnology Co., Ltd. (Benxi, China; certificate NO. SCXK (Liao) 2020–0001) and housed in a specific pathogen-free environment under a 12-h light/dark cycle at 25 °C and 40%–60% humidity with free access to food and water. After a 1-week acclimatization period, the rats were randomly assigned to four groups (n = 6): a blank control group, which received normal saline; a SF-AR herb pair group, which was administered SF-AR herb pair at 10.0 g/kg/d; an SF alone group, receiving SF at 7.5 g/kg/d; and an AR alone group, receiving AR at 2.5 g/kg/d. The treatment lasted for 2 weeks. Plasma samples were collected from the rats prior to the first administration as blank controls, and subsequent plasma samples were collected 0.5, 2.0, and 4.0 h after the final dose. Retro-orbital blood was collected under isoflurane anesthesia (induction 3%–5% in oxygen via nose cone; maintenance 1%–2%) using heparinized capillaries, and the plasma samples from each group were pooled. Terminal procedures were conducted under deep isoflurane anesthesia followed immediately by decapitation. Death was confirmed by cessation of heartbeat and respiration and loss of corneal and pedal reflexes. All animal procedures complied with the AVMA Guidelines for the Euthanasia of Animals and ARRIVE. The organs of the rats, including hearts, livers, spleens, lungs, and kidneys, which were rapidly removed and collected, washed with normal saline, and stored at −80 °C for further analysis.

#### 2.4.2 Preparation of plasma and tissue samples

For plasma sample preparation, 3 mL of methanol was added to 1 mL of plasma, followed by vortex mixing for 3 min and centrifugation at 12,000 rpm for 5 min at 4 °C. The supernatant was then transferred and dried at 30 °C under a nitrogen flow. The residue was dissolved in 50 µL of methanol, vortexed for 3 min, sonicated for 10 min, and centrifuged at 12,000 rpm for 5 min at 4 °C. The plasma samples were then analyzed via LC-QTOF-MS.

For tissue sample preparation, approximately 50 mg of tissue was weighed, minced, and homogenized in 1 mL of normal saline on an ice bath for 30 s. The homogenate was then centrifuged at 4,000 rpm for 3 min at 4 °C. Homogenates from each group were pooled and mixed thoroughly. A 100 µL aliquot was taken, mixed with 300 µL of methanol, vortexed for 3 min, and centrifuged at 12,000 rpm for 10 min at 4 °C. The supernatant was transferred to a centrifuge tube, dried under a nitrogen stream, and reconstituted in 50 µL of a 50% methanol-water solution. The mixture was vortexed for 5 min, sonicated for 5 min, and centrifuged for 10 min at 12,000 rpm at 4 °C. The supernatant was collected for subsequent analysis.

### 2.5 LC-QTOF-MS analysis

The liquid chromatography separation and high-resolution mass spectrometry analysis were performed using an Agilent 1260 Infinity HPLC System (Agilent, Santa Clara, CA, United States) coupled with a Sciex TripleTOF 5600^+^ Quadrupole Time-of-Flight tandem mass spectrometer (Sciex, Framingham, MA, United States). Data acquisition was carried out using the Analyst TF software (version 1.7.1, Sciex).

The chromatographic separation was achieved on a Shim-pack GIST C18 (4.6 × 250 mm, 5 µm) column maintained at 30 °C. The analysis was conducted with a gradient elution using 10 mmol/L ammonium acetate in water (adjusted to pH 8.35 using ammonia solution) (A) - acetonitrile (B) over a total run time of 65 min. The gradient program was as follows: 95% A → 85% A at 0–10.0 min; 85% A → 79% A at 10.0–25.0 min; 79% A → 77% A at 25.0–35.0 min; 77% A → 40% A at 35.0–50.0 min; 40% A → 95% A at 50.0–55.0 min; followed by a 10-min hold at 95% A. The flow rate was set as 1 mL min^-1^, with a sample injection volume of 5.0 μL at 4 °C.

For mass spectrometry, the TOF MS full scan and data-dependent CID fragmentation scan (IDA mode) were conducted in both positive and negative electrospray ionization modes. The ion source parameters were set as follows: temperature, 600 °C; ion spray voltage, 5500 V (positive ion mode)/−4500 V (negative ion mode); nebulizer gas (Gas 1), 55 psi; heater gas (Gas 2), 55 psi; curtain gas, 35 psi; and declustering potential, 100 V (positive ion mode)/−100 V (negative ion mode). In IDA mode, dynamic background subtraction was enabled for optimal IDA coverage, with spectra exceeding 50 cps selected for eight dependent CID fragmentation scans. Isotopes within 4 Da were excluded, mass tolerance was set at 50 mDa, and collision energy was maintained at 45 V (positive ion mode)/−45 V (negative ion mode), with a collision energy spread of 15 V. One experimental period of 840 m consisted of nine experiments: the first being the TOF MS scan lasting 150 m, followed by 8 CID fragmentation scans of 80 m each. Nitrogen was used as both the nebulizer and auxiliary gas. The TOF MS full scan was performed within the mass range of *m/z* 100–1500, and the CID fragmentation scan within the mass range of *m/z* 80–1250.

### 2.6 Systematic characterization of constituents in SF-AR herb pair, plasma and organ tissue samples

The SF-AR herb pair, SF alone, AR alone extracts, as well as plasma and tissue samples, were analyzed by LC-QTOF-MS. The acquired data were processed using multiple data analysis approaches with Sciex PeakView® (version 2.2, Sciex) and MasterView™ (version 1.1, Sciex) software, as previously described. Specifically, first of all, to comprehensively analyze and characterize the chemical constituents in SF-AR, an in-house screening compound database was established for the potentially contained constituents in SF and AR. This database was created using LibraryView® (version 2.2, Sciex), incorporating data from previous literature and publicly available compound databases such as ChemSpider, SciFinder, and PubChem. During database creation, data from different plant parts, various processing methods, and plants of the same genus and family were included to ensure a comprehensive screening. Subsequently, high-resolution mass spectrometry was employed to analyze reference standard solutions of representative compounds, summarizing fragmentation patterns and characteristic ions for different compound classes. The screening database includes essential information such as the compound name, molecular formula, theoretical molecular mass, and typical MS fragmentation patterns. This database facilitates rapid screening and preliminary identification of target compounds in SF-AR.

The systematic compound characterization process was based on the accurate *m/z* of precursor ions (mass error <5.5 ppm), associated isotopic patterns, chromatographic retention behavior, and MS/MS fragmentation patterns. A comparative analysis with blank samples was conducted to identify and characterize any unanticipated constituents. Ions uniquely detected in the SF-AR herb pair sample, and not observed in the blank control, were extracted and subjected to detailed analysis using the formula finder module within MasterView software. A mass defect and fragment filtering approach, along with a formula prediction strategy, was also applied for comprehensive characterization, as previously described.

Following the comprehensive screening of constituents in SF-AR, the obtained information was compiled into a new SF-AR herb pair compound database. This database was used to match the MS data from rat plasma and organ tissue samples collected after oral administration, leading to the rapid identification of the absorbed constituents *in vivo* following SF-AR administration.

### 2.7 Exploration of anti-HCC mechanism of SF-AR herb pair using network pharmacology

The identified absorbed constituents in plasma of SF-AR herb pair were imported into the SwissTargetPrediction website (http://www.swisstargetprediction.ch/, access date: February, 2024) for the analysis of their potential therapeutic targets. Meanwhile, the HCC therapeutic targets were retrieved from GeneCards (version 5.19, https://www.genecards.org/), DisGeNET (version 24, https://www.disgenet.org/) and OMIM (https://www.omim.org/, access date: February, 2024). Subsequently, the therapeutic targets of the absorbed constituents were mapped to the HCC targets, and the obtained intersecting targets were imported into the DAVID Knowledgebase online tool (v2023q4, https://david.ncifcrf. gov) to explore relevant biological processes and pathways from the Gene Ontology (GO) and Kyoto Encyclopedia of Genes and Genomes (KEGG) databases, respectively. The significantly enriched potential key targets were uploaded to the STRING (version 12.0, https://cn.string-db.org) database to obtain protein-protein interaction (PPI) relationships. A key node identification algorithm was then applied to screen these potential targets, and the screened key targets were imported into Cytoscape software (version 3.8.2) for PPI network visualization and network analysis. Finally, a comprehensive network of “compounds-targets-pathways” was constructed to screen potential active constituents in SF-AR herb pair.

### 2.8 Molecular docking of potential key targets and characterized constituents

The three-dimensional structures of the potential key constituents and key proteins were obtained from the PubChem database (https://pubchem.ncbi.nlm.nih.gov) and the Protein Data Bank (PDB) (https://www.rcsb.org/), respectively. The PDB IDs for COX2, IKKβ and NF-κB p65 were 3LN1, 4KIK and 1VKX, respectively. Maestro software (Schrödinger, LLC, New York, United States) was employed to process the protein structures, including protonation state adjustments and dehydration steps to ensure that the ligand structures conformed to low-energy configurations. Semi-flexible molecular docking of the potential constituents with the three targets was performed using Maestro software (Schrödinger, LLC, New York, United States). The docking results were visualized with PyMOL software (version 2.4, NY, United States).

### 2.9 Statistical analysis

Data were analyzed using SPSS 17.0 for Windows (SPSS Inc.). All values are presented as means ± standard deviation (SD). Differences between experimental groups were evaluated using analysis of variance (ANOVA), followed by a t-test. A p-value of less than 0.05 was considered statistically significant.

## 3 Results

### 3.1 Inhibition of HCC cell growth by SF-AR herb pair

**FIGURE 2:**
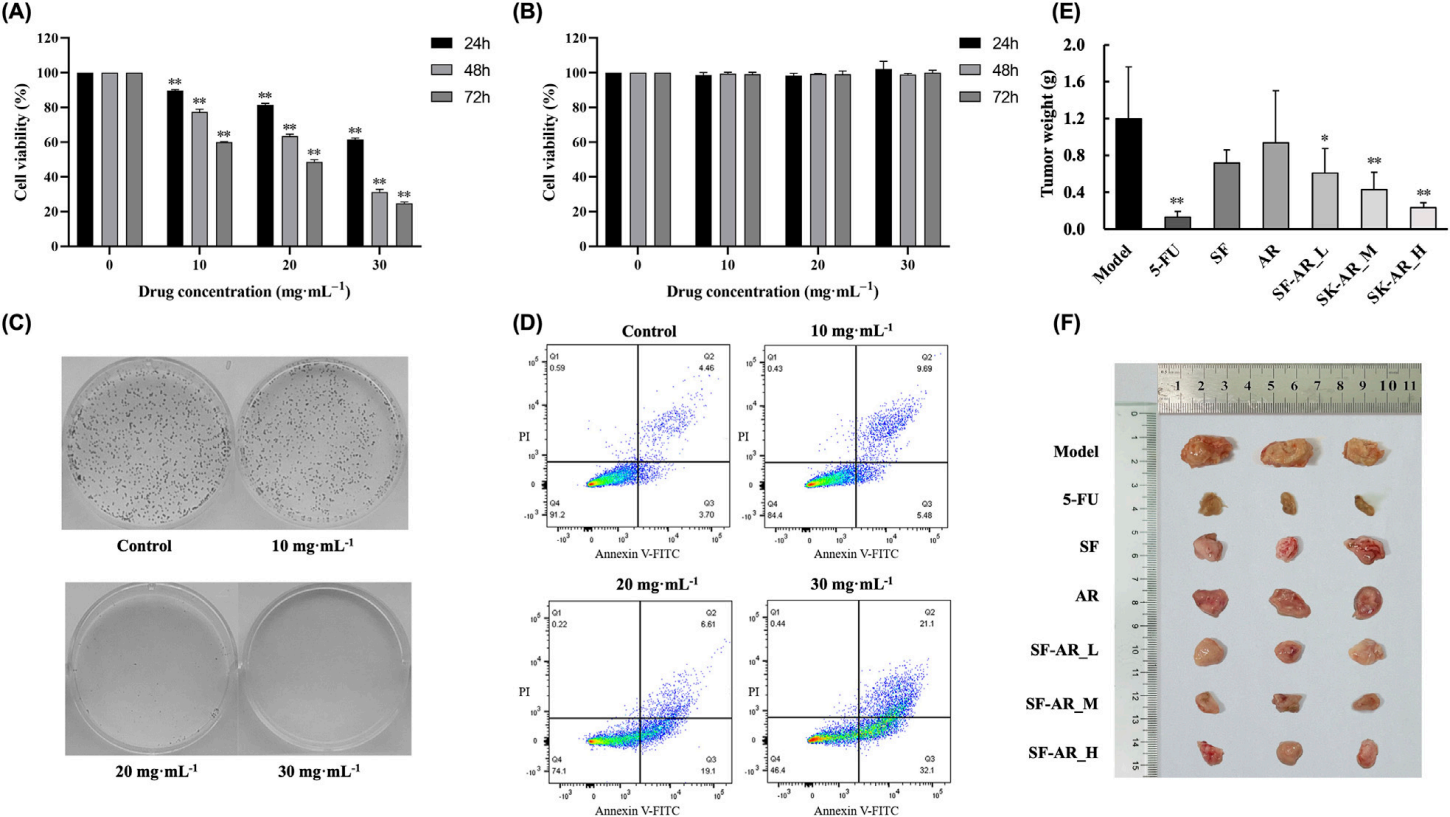
Anti-HCC effects of the SF-AR herb pair *in vitro* and *in vivo*. **(A)** Viability of HepG2 cells treated with various concentrations of SF-AR for 24, 48, and 72 h, assessed by MTT assay. **(B)** Viability of normal L02 hepatic cells treated with various concentrations of SF-AR for 24, 48, and 72 h, assessed by MTT assay. **(C)** Representative images of colony formation assays in HepG2 cells treated with SF-AR for 48 h. **(D)** Flow cytometry analysis of apoptosis in HepG2 cells treated with SF-AR for 48 h. **(E)** Tumor weights from H22 tumor allograft ICR mice at the end of the experiment. Mice were treated with vehicle (Model), 5-Fluorouracil (5-FU), SF alone (SF), AR alone (AR), or SF-AR herb pair at low (SF-AR_L), medium (SF-AR_M), and high (SF-AR_H) doses. **(F)** Representative images of excised tumors from each treatment group. Data in **(A,B)** are presented as mean ± SD (n = 3); **(E)** are presented as mean ± SD (n = 6). Data were analyzed by one-way ANOVA followed by a t-test. Statistical significance is denoted as ^*^ *p* < 0.05, ^**^ *p* < 0.01 compared to the control or model group.

To assess the anti-HCC efficacy of the SF-AR herb pair, its effects on the viability of human hepatocellular carcinoma HepG2 cells and normal human hepatic L02 cells were determined using the MTT assay following treatment with varying SF-AR concentrations over different durations. As illustrated in Figure 2A, SF-AR significantly inhibited HepG2 cell proliferation in both a dose- and time-dependent manner. Conversely, SF-AR did not impede the proliferation of normal L02 hepatic cells (Figure 2B), suggesting a selective cytotoxic effect against cancer cells.

The anti-proliferative capacity of SF-AR against HepG2 cells was further substantiated by colony formation assays (Figure 2C). While colony formation rates at lower SF-AR concentrations were comparable to those of the untreated control group, medium and high concentrations significantly reduced colony formation. Notably, no colonies were observed at the highest SF-AR concentration tested, indicating a potent suppression of HepG2 cell clonogenic survival.

Furthermore, apoptosis assays revealed that SF-AR induced apoptosis in HepG2 cells across all tested concentrations, with the extent of apoptosis increasing in a concentration-dependent manner (Figure 2D). This pro-apoptotic effect was markedly enhanced at higher drug concentrations; for instance, treatment with 30.0 mg/mL SF-AR resulted in apoptosis in over half of the HepG2 cell population. These collective findings suggest that the SF-AR herb pair exerts its anti-HCC effects, at least in part, by inhibiting proliferation and inducing apoptosis in liver cancer cells.

### 3.2 Inhibition of HCC tumor growth in mice by SF-AR herb pair

To evaluate the *in vivo* anti-HCC efficacy, H22 tumor cells were subcutaneously implanted into ICR mice, which were subsequently treated daily with oral administration of the SF-AR herb pair extract at various concentrations, or with SF or AR extracts alone. Throughout the 14-day treatment period, no significant differences in body weight were observed between the SF-AR treated groups and the model group, indicating that the oral administration of the herb pair was well-tolerated. As depicted in Figures 2E,F, all treatment regimens led to a reduction in tumor tissue weight compared to the untreated model group. Although administration of SF or AR alone resulted in some inhibition of tumor growth, these effects did not reach statistical significance relative to the model group. In stark contrast, both tumor weight and volume exhibited a dose-dependent decrease with increasing concentrations of the SF-AR herb pair, showing significant differences when compared to the model group. These data demonstrate that SF-AR treatment achieved more pronounced tumor suppression than either SF or AR administered individually, suggesting a enhanced anti-tumor effect of the herb pair.

Histopathological examination of tumor tissues via hematoxylin and eosin (H&E) staining further elucidated the anti-tumor effects (Supplementary Figure S1). In the model group, tumor sections revealed densely packed malignant cells with numerous mitotic figures. Conversely, tumors from SF-AR treated groups displayed a sparser arrangement of cells, reduced cellular density, and more extensive necrotic areas. Tumors from the positive control group (5-FU treated) showed less prominent mitotic figures, alongside multifocal necrosis, cytoplasmic vacuolization, and nuclear pyknosis or fragmentation. In mice treated with SF alone, AR alone, or low-dose SF-AR, only a limited number of mitotic figures and some granulocyte infiltrations were observed. Notably, the medium- and high-dose SF-AR groups exhibited markedly increased areas of tumor necrosis, characterized by karyolysis of necrotic tumor cells and minimal to absent inflammatory cell infiltration within the tumor stroma. Collectively, these results indicate that the SF-AR herb pair effectively inhibits HCC tumor growth *in vivo*.

### 3.3 Phytochemical profiling of the SF-AR herb pair by LC-QTOF-MS

The natural products within SF and AR are known for their substantial chemical diversity and complexity, primarily featuring flavonoids, alkaloids, and terpenoids. Alkaloids, mainly from SF, often present as structural isomers such as homologs, while flavonoids typically occur in both aglycone and glycosidic forms. Such structural intricacies pose considerable challenges for comprehensive constituent analysis using LC-MS. Therefore, meticulous optimization of LC-MS conditions is essential to achieve optimal chromatographic separation in these complex matrices and to minimize ion suppression arising from co-elution. In this study, both chromatographic and mass spectrometric parameters were rigorously optimized before sample analysis. This resulted in the successful development of an LC-QTOF-MS-based analytical method, which enabled the comprehensive characterization of constituents in the SF-AR herb pair, as well as the identification of those absorbed *in vivo*.

**FIGURE 3:**
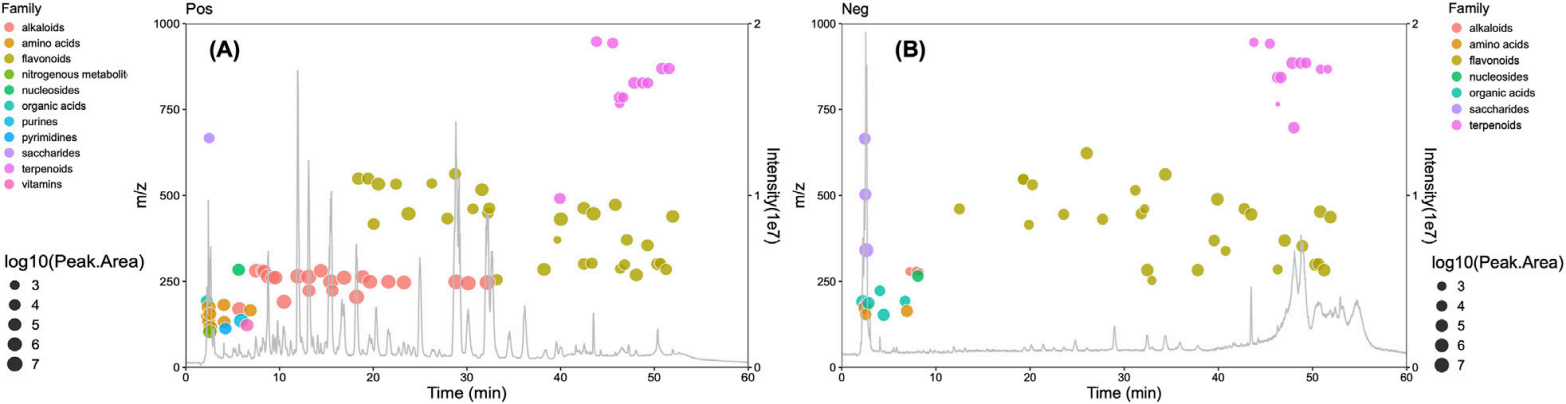
Phytochemical overview of the SF-AR herb pair extract analyzed by LC-QTOF-MS. The figure displays Total Ion Chromatograms (TICs) overlaid with bubble plots representing the 95 constituents identified in **(A)** positive ion mode (Pos) and **(B)** negative ion mode (Neg). Each bubble corresponds to a single identified compound. The x-axis indicates the retention time (min). The left y-axis shows the *m/z* of each compound. The right y-axis (Intensity) corresponds to the TIC. The size of each bubble is proportional to the log10 of its peak area.

The SF-AR herb pair extract was systematically profiled using an established MS data analysis strategy that integrated three key steps: database matching, structural relevance filtering, and untargeted compound screening. Through rigorous comparison of accurate mass-to-charge ratios (*m/z*) for precursor and product ions, chromatographic retention times, isotopic distributions, and fragmentation patterns, a total of 95 compounds were tentatively identified in the SF-AR extract. These included 37 flavonoids, 23 alkaloids, 12 terpenoids, five organic acids, 12 amino acids or nucleic acids, and six other compounds. The botanical origin of each characterized compound (SF, AR, or common to both) was determined by comparative analysis of data from SF-only and AR-only extracts. For all identified constituents, the mass accuracy of precursor ions was maintained within ±5 ppm. Detailed information regarding these compounds, including their source attribution, is presented in Supplementary Table S2. A comprehensive overview of the phytochemical profile of the SF-AR herb pair extract, displaying the distribution of all 95 identified compounds based on their retention time, *m/z*, peak area, and phytochemical class in both positive and negative ion modes, is presented in Figure 3.

#### 3.3.1 Identification of flavonoids in SF-AR herb pair

Flavonoids, widely present in both SF and AR, can be further classified into flavonoids, isoflavonoids, flavonols, etc., based on their structural characteristics. The results showed a total of 37 flavonoids were preliminarily characterized in SF-AR herb pair. Among these, 12 were common to both SF and AR, with isoflavonoids representing the predominant type. Additionally, 10 flavonoids were specific to SF, and 13 were unique to AR. In herbs, flavonoids commonly occur as glycosides coexisting with their corresponding aglycones, which are often observed in the CID MS spectra of glycosides. Due to the structural similarity of their core skeletons, flavonoid aglycones exhibit comparable CID fragmentation patterns. These patterns frequently include characteristic fragment ions, such as those resulting from Retro-Diels-Alder (RDA) cleavage at the C ring of the flavonoid skeleton, as well as neutral losses of CO, H_2_O, and C_2_H_2_O. By analyzing the fragmentation patterns of flavonoids, characteristic fragment ions and neutral losses were summarized. These features can be utilized within the data mining strategy of structural relevance filtering implemented in this study. This approach enables more comprehensive discovery of such constituents in the SF-AR herb pair and facilitates structural analysis of previously unidentified flavonoid.

**FIGURE 4:**
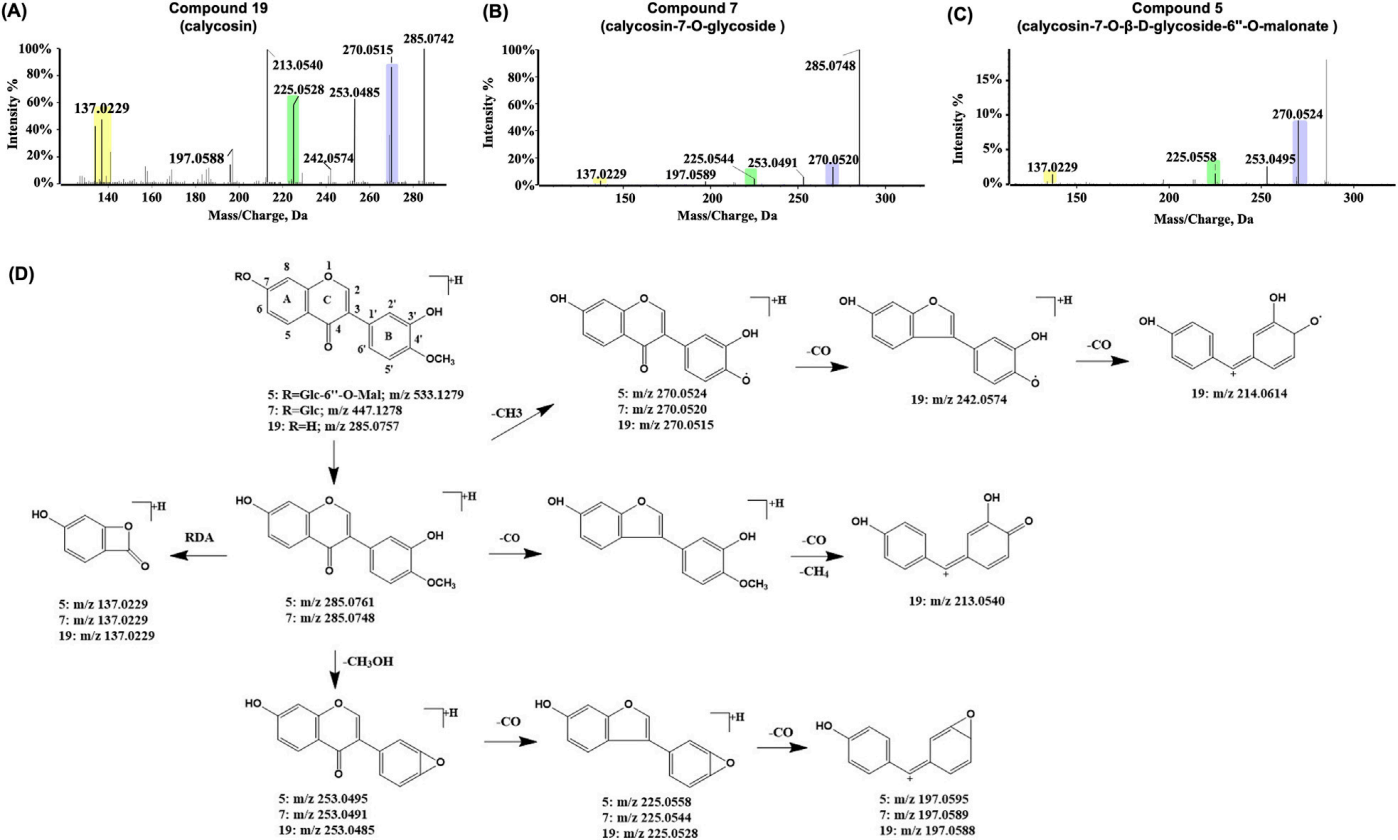
Representative MS/MS spectra and proposed fragmentation pathways for calycosin and related flavonoids from the SF-AR herb pair. MS/MS spectrum of **(A)** Compound 19 (calycosin), **(B)** Compound 7 (calycosin-7-O-glycoside) and **(C)** Compound 5 (calycosin-7-O-β-D-glycoside-6″-O-malonate). **(D)** Proposed MS/MS fragmentation pathways for Compounds 19, 7, and 5.

Calycosin and calycosin glycosides are representative isoflavonoids commonly found in both SF and AR. Their fragmentation patterns serve as reference models for characterizing other structurally similar isoflavonoid constituents in SF-AR. Consequently, these two compounds were selected to elucidate the characteristic fragmentation patterns of isoflavonoids. Compound 19 (Figure 4A), with a retention time of 38.23 min, generated a precursor ion of [M + H]^+^ with *m/z* of 285.0757 in the positive ion mode. CID analysis revealed a sequential loss of CH_3_, CH_3_OH, and multiple CO to form different characteristic fragment ions, such as *m/z* 270.0515 [M + H-CH_3_]^+^, *m/z* 253.0485 [M + H-CH_3_OH]^+^, *m/z* 225.0528 [M + H-CH_3_OH-CO]^+^, and *m/z* 197.0588 [M + H-CH_3_OH-2CO]^+^. In addition, RDA cleavage at the C ring produced a characteristic ion at *m/z* 137.0229. Compound 19 was characterized as calycosin (C_16_H_12_O_5_) by comparison of retention time with the reference standard and the established strategy for constituent characterization. Compound 7 (Figure 4B) had a retention time of 23.76 min and was also confirmed as calycosin-7-O-glycoside (C_22_H_22_O_10_), by retention time comparison with the reference standard. Comparing the chemical structures of compounds 19 and 7, calycosin is linked to a glucosyl residue at the 7-hydroxyl position, forming calycosin-7-O-glucoside. In the CID spectrum, the precursor ion [M + H]^+^ of the calycosin glycoside generates an [aglycone + H]^+^ ion with an *m/z* of 285.0748, along with fragment ions that were identical to calycosin at *m/z* 270.0520, m*/z* 225.0544 and *m/z* 137.0229. A detailed analysis of the characteristic product ions of the calycosin glycoside and its aglycone facilitates the identification of additional chromatographic peaks that exhibit similar fragmentation patterns and cleavage pathways.

Compound 5 (Figure 4C) had a retention time of 20.57 min, and in the positive ion mode, it generated a precursor ion of [M + H]^+^ with *m/z* of 533.1279. In CID, the precursor ion underwent a neutral loss of a saccharide residue (248 Da), yielding an aglycone ion at *m/z* 285.0761 ([aglycone + H]^+^). This fragmentation also produced a series of characteristic ions similar to those observed for calycosin, including *m/z* 270.0524 [aglycone + H-CH_3_]^+^, *m/z* 253.0495 [aglycone + H-CH_3_OH]^+^, *m/z* 225.0558 [aglycone + H-CH_3_OH-CO]^+^, and *m/z* 197.0595 [aglycone + H-CH_3_OH-2CO]^+^, as well as fragment ions identical to the ^1,3^A characteristic ion produced by RDA cleavage occurring in calycosin (*m/z* 137.0229). Based on the detected accurate *m/z* of the ions, the chemical formula of compound 5 is characterized to be C_25_H_24_O_13_. Given that the exact *m/z* of its aglycone ion closely corresponds to that of calycosin, it is inferred that the aglycone is calycosin, with fragment ions identified as [M-C_6_H_10_O_5_-C_3_H_2_O_3_+H]^+^. Since the glycosidic substitution in calycosin derivatives typically occurs at the C_7_ position of A ring, therefore, by integrating the fragmentation pattern interpretation with evidence from the literature, compound 5 is proposed to be calycosin-7-O-β-D-glycoside-6″-O-malonate (Lin et al., 2000; Zhang et al., 2015) (Figure 4D).

**FIGURE 5:**
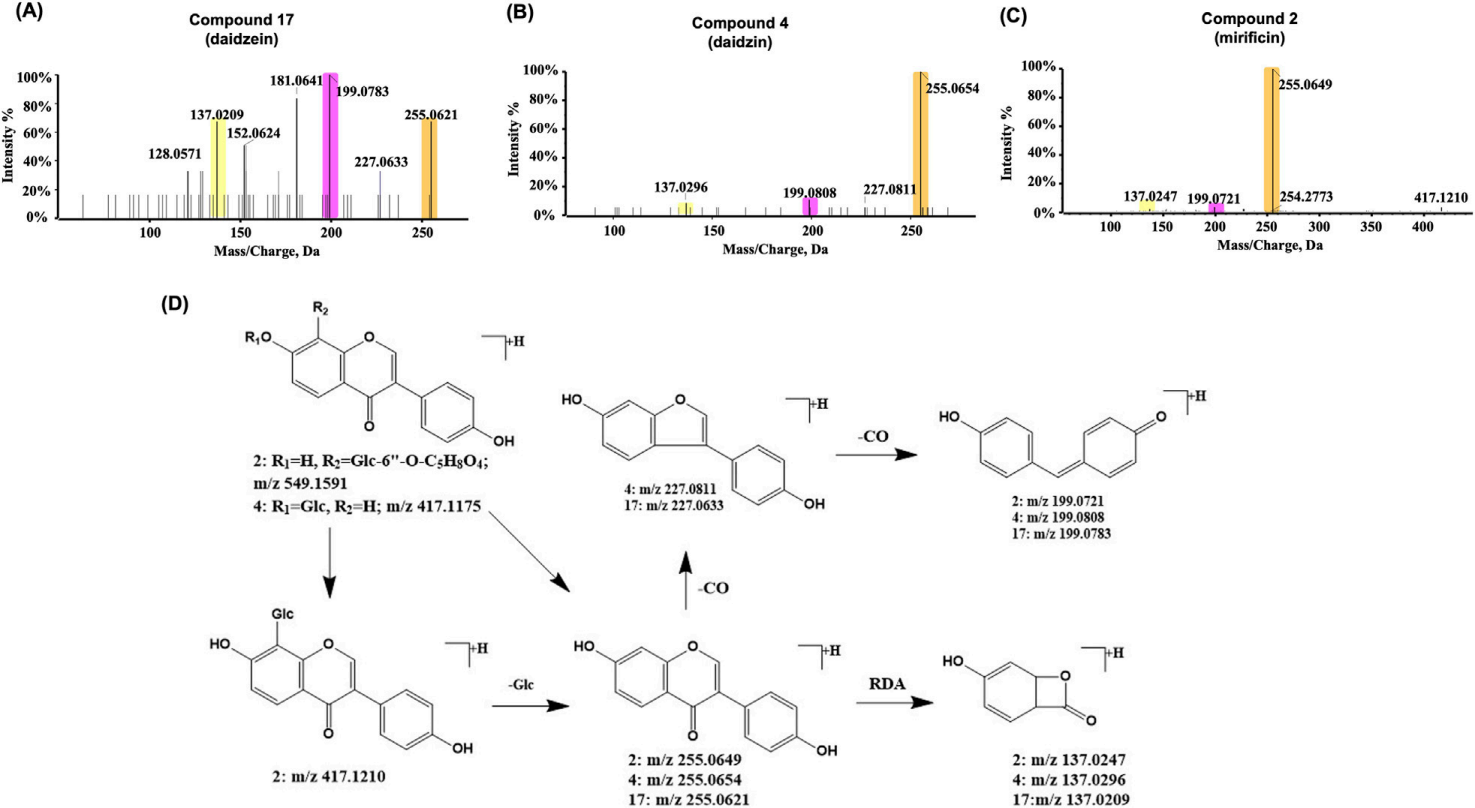
Representative MS/MS spectra and proposed fragmentation pathways for daidzein and its derivatives from the SF-AR herb pair. **(A)** MS/MS spectrum of Compound 17 (daidzein). **(B)** MS/MS spectrum showing fragmentation of the aglycone moiety ([Aglycone + H]^+^) derived from Compound 4 (daidzin). **(C)** MS/MS spectrum showing fragmentation of the aglycone moiety ([Aglycone + H]^+^) derived from Compound 2 (mirificin). **(D)** Proposed MS/MS fragmentation pathways for Compounds 17, 4, and 2.

Compound 17 (Figure 5A) was exclusively characterized in the SF-AR herb pair, exhibiting an [M + H]^+^ ion at *m/z* 255.06537 (C_15_H_10_O_4_) with a retention time of 33.19 min. In the CID spectra, a fragment ion at *m/z* 137.0209 was observed, matching that generated by the RDA cleavage of the calycosin C-ring. This finding indicates that the A and C rings of Compound 17 share structural similarity with calycosin, whereas potential differences likely reside in the B ring.

Additionally, Compound 17 yielded characteristic ions at *m/z* 227.0633 ([M + H−CO]^+^) and *m/z* 199.0783 ([M + H−2CO]^+^), corresponding to the B-ring cleavage fragments of calycosin ([M + H−CH_3_−CO]^+^ and [M + H−CH_3_−2CO]^+^, respectively), each differing by 15 Da (Figure 4D). Therefore, it was inferred that the structural distinction between compound 17 and calycosin lies in the absence of a methoxy group on the B ring. By integrating these observations with literature comparisons, peak 17 was characterized as daidzein (Tang et al., 2016). Glycosides and aglycones typically occur as paired compounds in herbs. Further analysis identified compound 4 (Figure 5B) (Tang et al., 2016; Xu et al., 2011), which exhibited an [M + H]^+^ at *m/z* 417.1175 and a retention time of 20.05 min. Upon the neutral loss of 162 Da, an [aglycone + H]^+^ was generated at *m/z* 255.0621, with fragment ions identical to those observed for compound 17. Based on these results, compound 4 is likely a glycoside of compound 17, conjugated with a saccharide, which is proposed to be daidzin, with the molecular formula C_21_H_20_O_9_.

Compound 2 (Figure 5C) was exclusively detected in SF-AR herb pair as well. In positive ion mode, the [M + H]^+^ ion of compound 2 appeared at *m/z* 549.1591, which underwent neutral loss of a pentose residue, apiose (132 Da), to yield a fragment ion at *m/z* 417.1210 ([M + H-apiose]^+^). This ion further lost a glucose residue (162 Da) to generate a fragment ion at *m/z* 255.0649 ([M + H-apiose-glucose]^+^). Subsequent fragmentation produced characteristic ions at *m/z* 199.0721 ([M + H-apiose-glucose-2CO]^+^), and *m/z* 137.0247 (^1,3^A RDA cleavage), consistent with the fragmentation pattern of compound 17. Based on these results, it was inferred that compound 17 is the aglycone of compound 2, with the [M + H-apiose-glucose]^+^ arising from the precursor ion ([M + H]^+^) losing the entire saccharide chain. The saccharide chain is composed of a hexose and a pentose, with the hexose attached to the aglycone. Thus, compound 2 is speculated to be mirificin (Weng et al., 2018), of (C_26_H_28_O_13_), a dihydroxyflavone and a flavone C-glycoside (Figure 5D).

**FIGURE 6:**
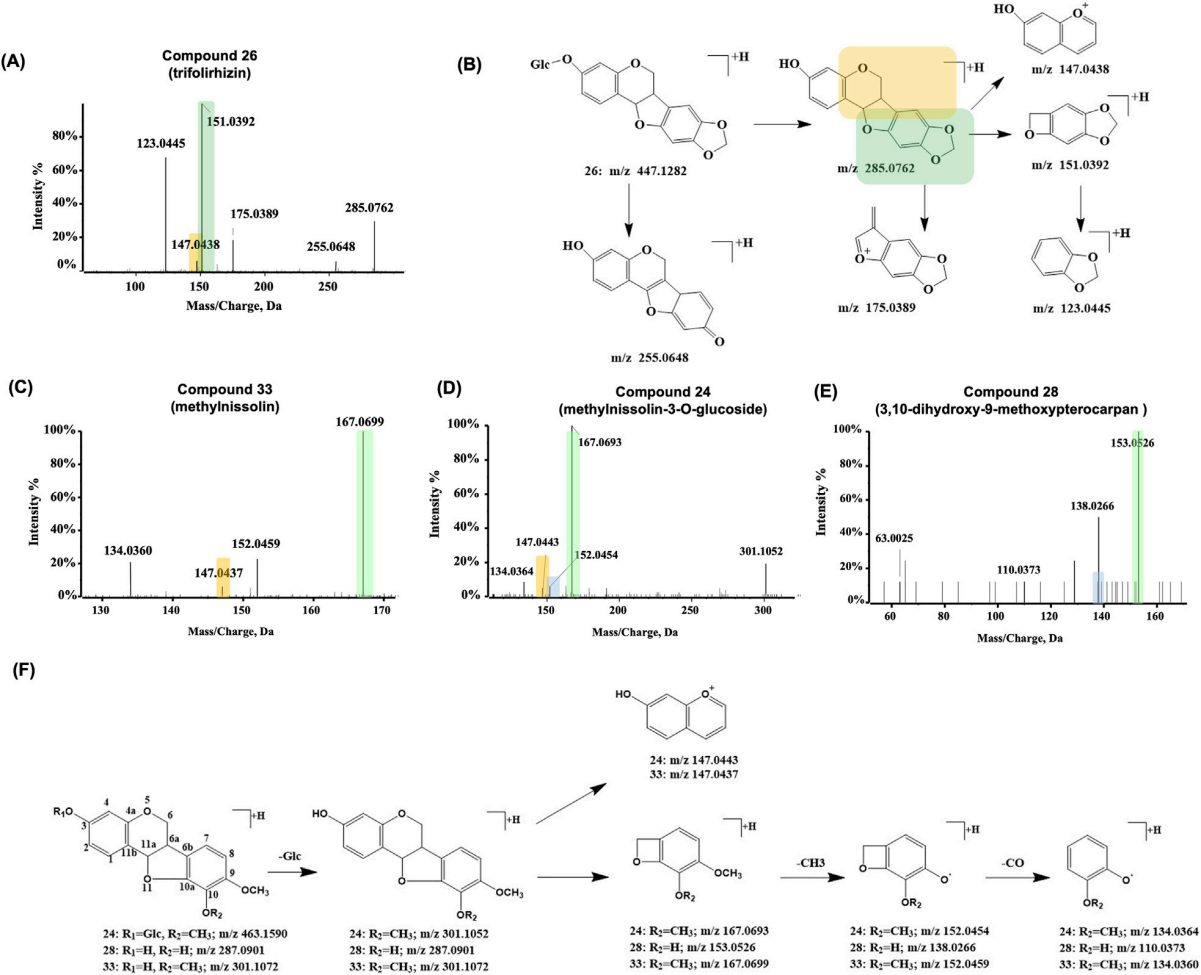
Representative MS/MS spectra and proposed fragmentation pathways for pterocarpan-type flavonoids from the SF-AR herb pair. **(A)** MS/MS spectrum of Compound 26 (trifolirhizin). **(B)** Proposed MS/MS fragmentation pathway for Compound 26 (trifolirhizin). MS/MS spectrum of **(C)** Compound 33 (methylnissolin), **(D)** Compound 24 (methylnissolin-3-O-glucoside) and **(E)** Compound 28 (3,10-dihydroxy-9-methoxypterocarpan). **(F)** Proposed general MS/MS fragmentation pathways for pterocarpans, illustrated with Compounds 24, 28, and 33.

Polycyclic flavonoid compounds were found in both SF and AR. Compound 26 (Figures 6A,B) with a retention time of 43.3 min was characterized as a typical pterocarpan compound, trifolirhizin (Yang et al., 2013) (C_22_H_22_O_10_), with [M + H]^+^ observed at *m/z* 447.1282. The compound consists of a 1-benzofuran moiety fused to a 2H-chromene moiety. In the CID spectrum, the precursor ion initially formed an aglycone ion at *m/z* 285.0762 ([M + H-Glc]^+^) and a further fragment at *m/z* 255.0648 ([M + H-Glc-CH_2_O]^+^). The aglycone ion underwent furan ring cleavage, yielding a benzo-pyrano structure at *m/z* 147.0438 ([M + H-Glc -C_7_H_6_O_3_]^+^) and *m/z* 151.0392 ([M + H-Glc -C_8_H_6_O_2_]^+^). The ion, with a quaternary heterocyclic structure, was unstable and underwent further fragmentation to generate *m/z* 123.0445 ([M + H-Glc -C_8_H_6_O_2_-CO]^+^). Upon cleavage of the pyran ring, a fragment at *m/z* 175.0389 ([M + H-Glc -C_6_H_6_O_2_]^+^) was observed. Due to the common substitution patterns on the 2H-chromene moiety, the benzo-pyrano structure at *m/z* 147.04 was identified as a characteristic fragment. By filtering of the fragment *m/z* 147.04, it was found that compounds 24 and 33 were in accordance with the same fragmentation pattern.

Compound 33 was detected at a retention time of 50.29 min, exhibiting an [M + H]^+^ ion at *m/z* 301.1072 (C_17_H_16_O_5_). As shown in Figure 6C, its fragmentation pattern generated ions at *m/z* 147.0437 ([M + H-C_8_H_10_O_3_]^+^) and *m/z* 167.0699 ([M + H-C_8_H_6_O_2_]^+^). Comparison of these furan ring cleavage fragment ions with those from compound 26 suggests both compounds share a benzo-pyrano core structure. Furthermore, an additional CH_4_ substituent on the 2H-chromene moiety of compound 33 indicates it has one more methyl group compared with the aglycone of compound 26. Based on other literature, compound 33 was proposed to be methylnissolin (Lin et al., 2000; Zhang L. et al., 2007; Zhang X. et al., 2007).

Compound 24 (Figure 6D) was detected at a retention time of 42.48 min, exhibiting an [M + H]^+^ ion at m/z 463.1590, consistent with the molecular formula C_23_H_26_O_10_. A neutral loss of 162 Da (glucose residue) yielded a fragment ion at *m/z* 301.1052, which matched the precursor ion of compound 33. Because the other fragment ions were also essentially identical for both compounds, compound 33 was inferred to be the aglycone of compound 24. Based on comparisons with other literature, compound 24 was preliminarily identified as methylnissolin-3-O-glucoside. Compound 28 showed a retention time of 46.2 min, with a precursor [M + H]^+^ ion at *m/z* 287.0901, which was 14 Da lower than that of compound 33. Its fragment ions at *m/z* 153.0526 and 138.0266 were also 14 Da lower than the characteristic furan-ring cleavage fragments of compound 33. These observations suggest that the substituent on the benzo-pyrano structure in compound 28 (Figure 6E) is missing a CH_2_ group relative to compound 33. By consulting the literature, compound 28 was proposed to be 3,10-dihydroxy-9-methoxypterocarpan. The fragmentation patterns of these three representative pterocarpans are shown in Figure 6F.

#### 3.3.2 Identification of alkaloids in SF-AR herb pair

In the SF-AR herb pair, a total of 23 alkaloids were tentatively identified, matching those previously detected in SF alone, indicating that the alkaloid profile in the herb pair is derived from SF. The alkaloids in SF mainly comprise matrine-, cytisine-, and spartenine-type alkaloid. Among these, matrine-type alkaloids predominate and are classified as quinolizidine alkaloids formed by the fusion of two quinazidine rings. Under positive ion mode, these compounds produce numerous characteristic fragment ions. Consequently, summarizing the fragmentation patterns of SF alkaloids is crucial for addressing the challenges posed by the extensive array of homologues and isomers present in SF.

Both compounds 50 and 58 produced a precursor ion at *m/z* 249.1960 ([M + H]^+^) in positive ion mode, consistent with the molecular formula C_15_H_24_N_2_O, and were observed at retention times of 15.5 min and 28.8 min, respectively. Comparison of their retention times and MS/MS spectra with those of reference standards confirmed the two compounds as sophoridine and matrine, respectively, both of which are matrine-type alkaloids. Previous studies have largely attributed their characteristic fragment ions to cleavage of the N16-C17 and C7-C11 bonds within the C-ring (Dong et al., 2021; Mei-Feng et al., 2019). However, some evidence suggests that proton transfer may induce cleavage at the N1-C6 bond, followed by a series of neutral losses (Zhang et al., 2023).

**FIGURE 7:**
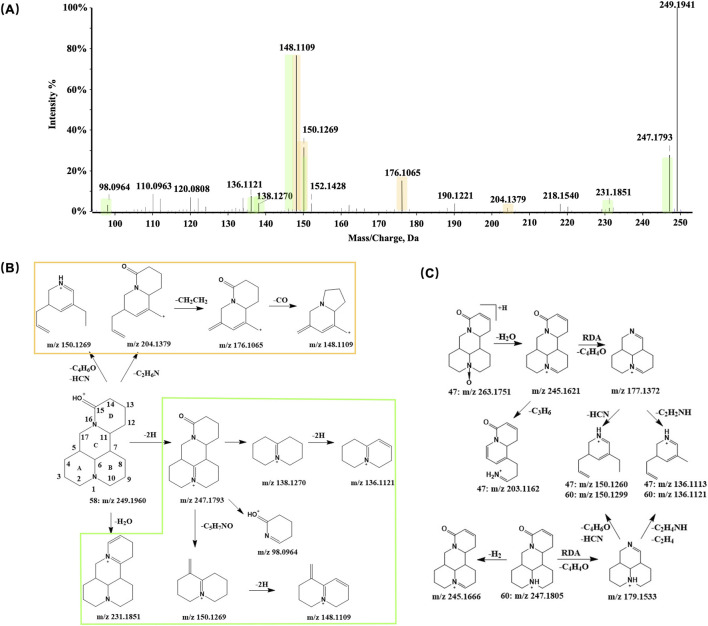
Representative MS/MS spectrum and proposed fragmentation pathways for matrine-type and related alkaloids from the SF-AR herb pair. **(A)** MS/MS spectrum of Compound 58 (matrine). **(B)** Proposed MS/MS fragmentation pathways for Compound 58. **(C)** Proposed MS/MS fragmentation pathways for Compound 47 (oxysophocarpine) and Compound 60 (sophocarpine).

In this study, alongside the characteristic fragment ions produced by the first cleavage pathway, multiple intermediate ions arising from the alternative cleavage route were also observed in the CID spectra of these two compounds. In particular, a fragment at *m/z* 204.13, formed by cleavage of the A and B rings, and a characteristic ion at *m/z* 148.11, generated through successive fragmentations, were detected. These results suggest that both cleavage pathways can occur for matrine-type alkaloids in positive ion mode, yielding fragment ions at *m/z* 150.12 and *m/z* 148.11 with high intensity in CID. Consequently, these ions may serve as diagnostic markers for matrine-type alkaloids. The proposed fragmentation pathways of compound 58 are shown in Figures 7A,B.

In addition to compounds 50 and 58, other compounds with fragment at *m/z* 249.19 were observed in the extracted ion chromatogram (EIC). Among these, compound 55, eluting at 19.65 min, produced major fragment ions at *m/z* 84.0820, 98.0969, 136.1118, 166.1221, and 206.1533 in CID. Notably, the ions at *m/z* 136.11 and m/z 166.12 are characteristic of spartenine-type alkaloids, generated by cleavage of the C6-C7 and C9-C10 bonds on ring B, and the C9-C11 and C16-C17 bonds on ring C, respectively. The fragment at *m/z* 98.0969 results from cleavage of the C9-C11 and C7-C17 bonds on ring C, indicating the absence of substituents on ring D. Based on these observations, compound 55 was tentatively identified as lupanine (Hwang et al., 2020; Li et al., 2022).

Analogous to the common co-occurrence of flavonoid glycosides and their corresponding aglycones, both tertiary amine alkaloids and their N-oxide forms are typically detected in SF. Under reversed-phase chromatographic conditions, the oxidized alkaloids usually exhibit higher hydrophilicity and thus may be eluted with shorter retention times. In CID, oxidized alkaloids initially undergo the neutral loss of H_2_O, yielding [M + H-H_2_O]^+^ ions that contain an additional double bond compared to their reduced counterparts. These ions subsequently produce the same fragments as their reduced forms. For example, compound 46, eluting at 11.96 min, was identified as oxymatrine (C_15_H_24_N_2_O_2_), the N-oxide of matrine, based on its fragmentation pattern and comparison with a reference standard. Its precursor [M + H]^+^ ion at *m/z* 265.1908 first lost H_2_O, forming the [M + H-H_2_O]^+^ at *m/z* 247.1787. Further fragmentation produced ions at *m/z* 150.12 ([M + H-H_2_O-C_4_H_6_O-HCN]^+^) and *m/z* 176.10 ([M + H-H_2_O-C_2_H_4_NH-C_2_H_4_]^+^), mirroring the characteristic fragments of matrine.

Employing the established characterization strategy and reference standards, compound 60 was confirmed as sophocarpine (C_15_H_22_N_2_O), with retention time of 32.17 min. Structurally, sophocarpine closely resembles matrine, differing only by an additional double bond in the D-ring. RDA cleavage of sophocarpine’s D-ring produces a characteristic fragment ion at *m/z* 179.1533. Compound 47 exhibited a precursor ion [M + H]^+^ at *m/z* 263.1751. Under CID, initial bond cleavage between the tertiary amine nitrogen and oxygen led to the neutral loss of H_2_O, generating [M + H-H_2_O]^+^ at *m/z* 245.1621. A subsequent RDA cleavage (loss of C_4_H_4_O) yielded the fragment at *m/z* 177.1378, which could further lose CHN or C_2_H_4_NH to form fragment at *m/z* 150.126 or *m/z* 136.1113, respectively. By comparing these results with a reference standard, compound 47 was identified as oxysophocarpine (C_15_H_22_N_2_O_2_), the N-oxide of sophocarpine (Figure 7C).

#### 3.3.3 Identification of terpenoids and other compounds in SF-AR herb pair

Compared with SF, AR contains more types of terpenoids, including astragaloside IV, which is commonly used as a key quality marker of AR. In total, 12 terpenoids were identified in SF-AR herb pair, the majority of which originate from AR. Under typical mass spectrometric conditions, triterpenoids often generate [M + H]^+^ or [M + AcO-H]^−^ precursor ions in positive and negative ion modes, respectively. In CID, functional groups such as hydroxyl or carboxyl groups on the triterpene skeleton typically undergo neutral loss of H_2_O or CO_2_, yielding the corresponding fragment ions.

**FIGURE 8:**
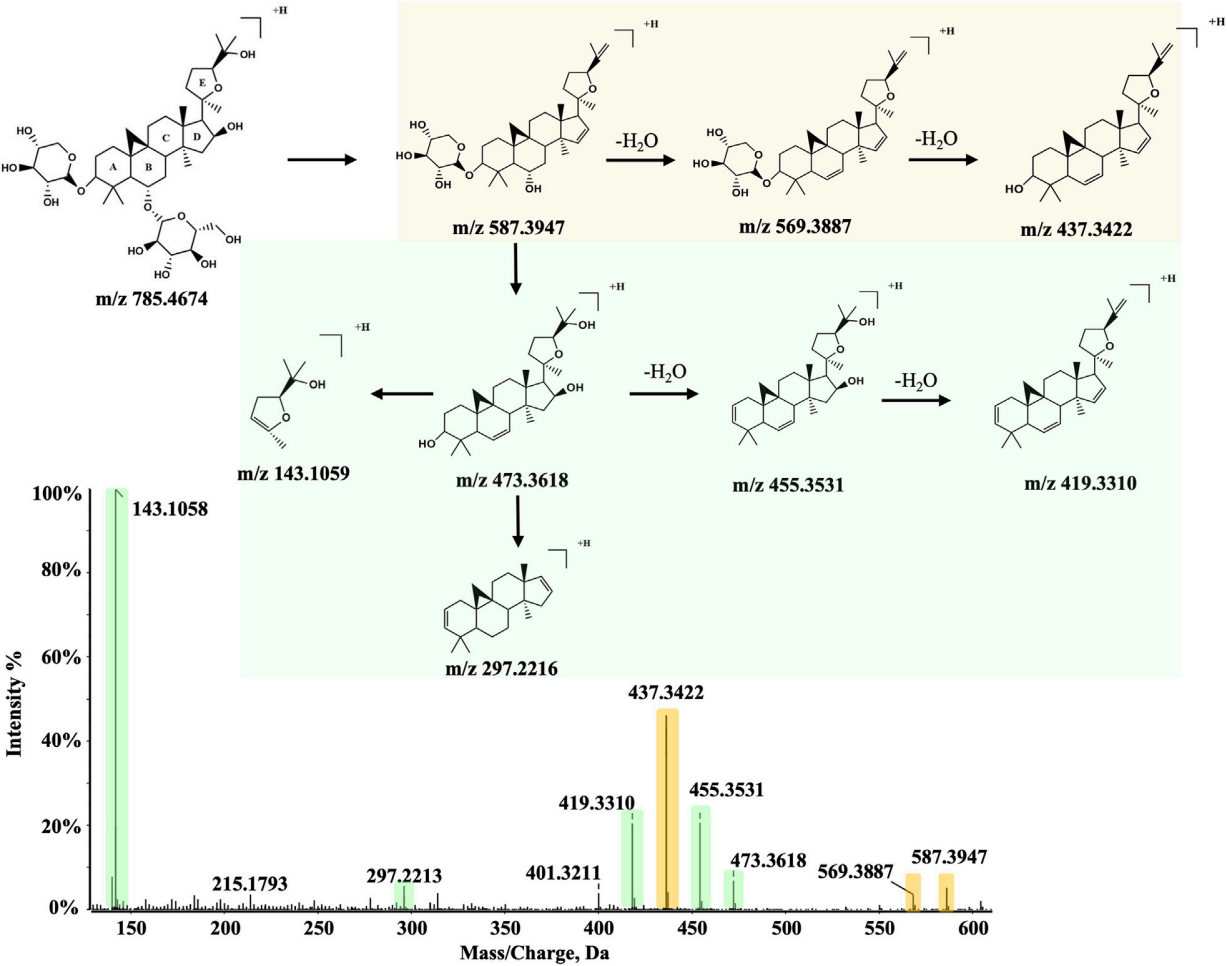
Representative MS/MS spectrum and proposed fragmentation pathway for Compound 65 (astragaloside IV) from the SF-AR herb pair.

Compound 65 (Figure 8), with a retention time of 46.3 min, was detected in both positive and negative ion modes, exhibiting precursor ions at *m/z* 785.4674 ([M + H]^+^) and *m/z* 843.4741 ([M + AcO-H]^−^). These observations are consistent with a molecular formula of C_41_H_68_O_14_. By following the established analytical strategy and comparing with a reference standard, compound 65 was identified as astragaloside IV. In the positive ion mode, the [M + H]^+^ undergoes sequential neutral losses, due to the multiple hydroxyl groups in its structure. Specifically, neutral loss of one glucose residue and two H_2_O produces the fragment [M + H-glc-2H_2_O]^+^ at *m/z* 587.3947. Additional water loss and cleavage of a xylose residue generate a series of fragment ions: [M + H-glc-3H_2_O]^+^ at *m/z* 569.3887, [M + H-Glc-3H_2_O-xyl]^+^ at *m/z* 437.3422, and [M + H-glc-4H_2_O-xyl]^+^ at *m/z* 419.3310. Following the loss of both glucose and xylose residues, astragaloside IV forms the fragment [M + H-glc-xyl-H_2_O]^+^ at *m/z* 473.3618. Cleavage of the C17-C18 bond (which links rings D and E) yields fragment ions at *m/z* 143.1059 and *m/z* 297.2216. The fragments observed at *m/z* 455.35, 437.34, and 419.33 arise from the sequential loss of H_2_O from the *m/z* 473.36. Using a similar approach and comparison with a reference standard, compound 67 was identified as astragaloside II, which follows an analogous fragmentation pathway.

Compound 63, a constituent which is present in both SF and AR, eluted at 45.56 min and produced a precursor ion [M + H]^+^ at *m/z* 943.5239 in the positive ion mode, corresponding to the C_48_H_78_O_18_. Under CID, the precursor ion successively lost groups linked via glycosidic bonds. Initially, rhamnose and one H_2_O were lost, producing fragments at *m/z* 797.4538 ([M + H-C_6_H_12_O_4_]^+^ and *m/z* 781.4714 ([M + H-C_6_H_12_O_4_-H_2_O]^+^). Subsequently, galactose and additional H_2_O were eliminated to yield *m/z* 617.3956 ([M + H-C_6_H_12_O_4_-C_6_H_12_O_6_]^+^) and *m/z* 599.3944 ([M + H-C_6_H_12_O_4_-C_6_H_12_O_6-_H_2_O]^+^). Finally, glycosidic bond cleavage and dehydration of the aglycone produced ions at *m/z* 441.3739 ([Aglycone + H-H_2_O]^+^), *m/z* 423.3611 ([Aglycone + H-2H_2_O]^+^), and *m/z* 405.3532 ([Aglycone + H-3H_2_O]^+^). Based on comparisons with published data, compound 63 was tentatively identified as soyasaponin I (Gu et al., 2002; Pan et al., 2022; Wang et al., 2019), an oleanane-type triterpenoid with hydroxy substituents at the C-3, C-22, and C-24 positions. In the MS/MS spectrum, *m/z* 305.0863 (C_23_H_29_) arises from cleavage of the oleanane A-ring and the loss of CH_4_ from ring E, introducing two double bonds. The ion at *m/z* 221.1960 (C_17_H_17_) is generated by further fragmentation of *m/z* 305.0863 through cleavage of E-ring, coupled with the loss of isobutane (C_4_H_8_) and methyl groups at C-8 and C-14, resulting in two double bonds in the aglycone core.

In addition to the above, five organic acids, twelve amino or nucleic acids, and six other types of compounds were also tentatively identified in SF-AR herb pair. Preliminary characterization of various saccharides further facilitated elucidation of glycosidic chains in SF-AR glycosides.

### 3.4 Identification of absorbed constituents of SF, AR, and SF-AR herbal pair in rat plasma and tissues after administration

A new SF-AR herbal pair constituent database was established using detailed information on the accurate mass of precursor and fragment ions, isotopic patterns, and chromatographic retention behaviors for 95 characterized constituents. To minimize interference from other substances in the biological matrix, ions detected in plasma and tissue samples following the administration of the herbal pair, SF, or AR, but absent or minimally responsive in the blank control group, were extracted and screened. Comprehensive constituent analysis of rat plasma and tissues after administration revealed the results summarized in Tables 1, 2. Notably, 22 prototype constituents were identified in the plasma after SF-AR herbal pair administration, including seven flavonoids, 14 alkaloids, and one organic acid. The flavonoids absorbed into the plasma were common to both SF and AR, while all alkaloids originated from SF. The single organic acid was also derived from SF. Terpenoids were not detected in the biological samples, likely due to their low concentration in the herb pair itself. Flavonoids and alkaloids were widely distributed in the liver, kidneys, and spleen following administration, suggesting that these two classes of compounds may be the primary active constituents in the SF-AR herb pair contributing to its anti-tumor effects.

**TABLE 1:** Prototype constituents identified in rat plasma following oral administration of the SF-AR herb pair, SF alone, and AR alone.

NO.	t_R_ (min)	Compound	Formula	Adduct	Mass error (ppm)		
					SF	AR	SF-AR
1	23.76	Calycosin-7-O-Glc	C_22_H_22_O_10_	[M + H]^+^	−0.8	−4.8	−2.3
2	38.23	Calycosin	C_16_H_12_O_5_	[M + H]^+^	−3.1	−2.3	−0.9
3	40.43	Ononin	C_22_H_22_O_9_	[M + H]^+^	−0.7	−2.5	−3.6
4	42.34	Methylnissolin-3-O-Glc	C_23_H_26_O_10_	[M + H]^+^	-	−4.5	−3.1
5	43.34	Trifolirhizin	C_22_H_22_O_10_	[M + H]^+^	−0.1	-	0.4
6	48.08	Formononetin	C_16_H_12_O_4_	[M + H]^+^	2.1	−0.3	−0.9
7	49.20	Xanthohumol	C_21_H_22_O_5_	[M + H]^+^	−0.6	-	−2.4
8	8.30	Sophoranhol N-oxide	C_15_H_24_N_2_O_3_	[M + H]^+^	−1.1	0.2	−2.1
9	8.78	Isomer of Oxymatrine	C_15_H_24_N_2_O_2_	[M + H]^+^	0.0	−0.5	−2.5
10	10.47	Cytisine	C_11_H_14_N_2_O	[M + H]^+^	−2.2	-	−4.5
11	11.96	Oxymatrine	C_15_H_24_N_2_O_2_	[M + H]^+^	−1.0	-	−2.7
12	13.11	Oxysophocarpine	C_15_H_22_N_2_O_2_	[M + H]^+^	−0.8	-	−3.4
13	13.13	Kuraramine	C_12_H_18_N_2_O_2_	[M + H]^+^	−1.7	-	−2.2
14	15.51	Sophoridine	C_15_H_24_N_2_O	[M + H]^+^	−0.5	-	−4.2
15	16.89	Baptifoline	C_15_H_20_N_2_O_2_	[M + H]^+^	−1.0	-	−2.4
16	18.26	N-methyl cytisine	C_12_H_16_N_2_O	[M + H]^+^	−0.5	-	−1.8
17	19.65	Lupanine	C_15_H_24_N_2_O	[M + H]^+^	0.1	-	−5.2
18	21.62	Isolupanine	C_15_H_24_N_2_O	[M + H]^+^	−1.0	-	−3.7
19	23.57	7,11-Dehyromatrine	C_15_H_22_N_2_O	[M + H]^+^	−0.9	-	−2.3
20	28.84	Matrine	C_15_H_24_N_2_O	[M + H]^+^	−0.7	-	−2.4
21	32.34	Sophocarpine	C_15_H_22_N_2_O	[M + H]^+^	0.3	-	−3.0
22	4.50	Protocatechuic acid	C_7_H_6_O_4_	[M-H]^-^	−1.0	-	1.9

**TABLE 2:** Distribution of key absorbed constituents in rat tissues following oral administration of the SF-AR herb pair, SF alone, and AR alone.

NO.	Compound	Formula	Adduct	Liver Mass error (ppm)			Spleen Mass error (ppm)		
				(SF)	(AR)	(SF-AR)	(SF)	(AR)	(SF-AR)
1	Calycosin-7-O-Glc	C_22_H_22_O_10_	[M + H]^+^	-	-	−2.6	-	-	-
2	Daidzein	C_15_H_10_O_4_	[M + H]^+^	−1	−1.1	−3.6			-
3	Calycosin	C_16_H_12_O_5_	[M + H]^+^	−0.1	−2.8	−0.8	-	-	-
4	Ononin	C_22_H_22_O_9_	[M + H]^+^	−4.8	−2.8	−2.2	-	-	−2.7
5	Methylnissolin-3-O-Glc	C_23_H_26_O_10_	[M + H]^+^	-	0.3	-	-	-	-
6	Trifolirhizin	C_22_H_22_O_10_	[M + H]^+^	−1	-	−0.5	-	-	-
7	Formononetin	C_16_H_12_O_4_	[M + H]^+^	0.2	−0.9	−1	-	-	−1.4
8	Xanthohumol	C_21_H_22_O_5_	[M + H]^+^	−2.3	-	−0.7	-	-	-
9	Sophoranhol N-oxide	C_15_H_24_N_2_O_3_	[M + H]^+^	−1.3	-	−0.6	-	-	−0.8
10	Isomer of Oxymatrine	C_15_H_24_N_2_O_2_	[M + H]^+^	−1	-	−1.7	−1.6	-	−1.1
11	Cytisine	C_11_H_14_N_2_O	[M + H]^+^	−1.8	-	−1.8	-	-	-
12	Oxymatrine	C_15_H_24_N_2_O_2_	[M + H]^+^	−1.8	-	−1.7	−1.4	-	−2
13	Oxysophocarpine	C_15_H_22_N_2_O_2_	[M + H]^+^	−1	-	−1.7	−0.2	-	−2.2
14	Kuraramine	C_12_H_18_N_2_O_2_	[M + H]^+^	−2.5	-	1	-	-	−3.4
15	Sophoridine	C_15_H_24_N_2_O	[M + H]^+^	−2.1	-	−0.8	-	-	-
16	Baptifoline	C_15_H_20_N_2_O_2_	[M + H]^+^	−2.2	-	−0.6	−1.9	-	−2
17	N-methyl cytisine	C_12_H_16_N_2_O	[M + H]^+^	−1.4	-	−0.9	−1.5	-	−1.7
18	Lupanine	C_15_H_24_N_2_O	[M + H]^+^	−2.4	-	−0.1	-	-	-
19	Isolupanine	C_15_H_24_N_2_O	[M + H]^+^	−1.7	-	−1.2	-	-	-
20	7,11-Dehyromatrine	C_15_H_22_N_2_O	[M + H]^+^	−2	-	−1.2	-	-	-
21	Matrine	C_15_H_24_N_2_O	[M + H]^+^	−1	-	−0.6	-	-	-
22	Sophocarpine	C_15_H_22_N_2_O	[M + H]^+^	−0.8	-	−1.4	-	-	-
23	Protocatechuic acid	C_7_H_6_O_4_	[M-H]^-^	-	-	−0.8	-	-	-

### 3.5 Prediction of anti-HCC mechanisms of SF-AR herb pair using network pharmacology

The 22 prototype constituents identified in plasma were used for network pharmacology analysis to discover the anti-HCC mechanisms of the SF-AR herb pair. Utilizing the online database SwissTargetPrediction, 256 target proteins predicted from the absorbed constituents were obtained. Additionally, 2,173 targets were obtained by searching the HCC-related data in the GeneCards, DisGeNET, and OMIM databases. The intersection of these two sets yielded 95 common targets, which were considered primary therapeutic targets for the anti-HCC effects of the SF-AR herb pair and were used for further analysis.

**FIGURE 9:**
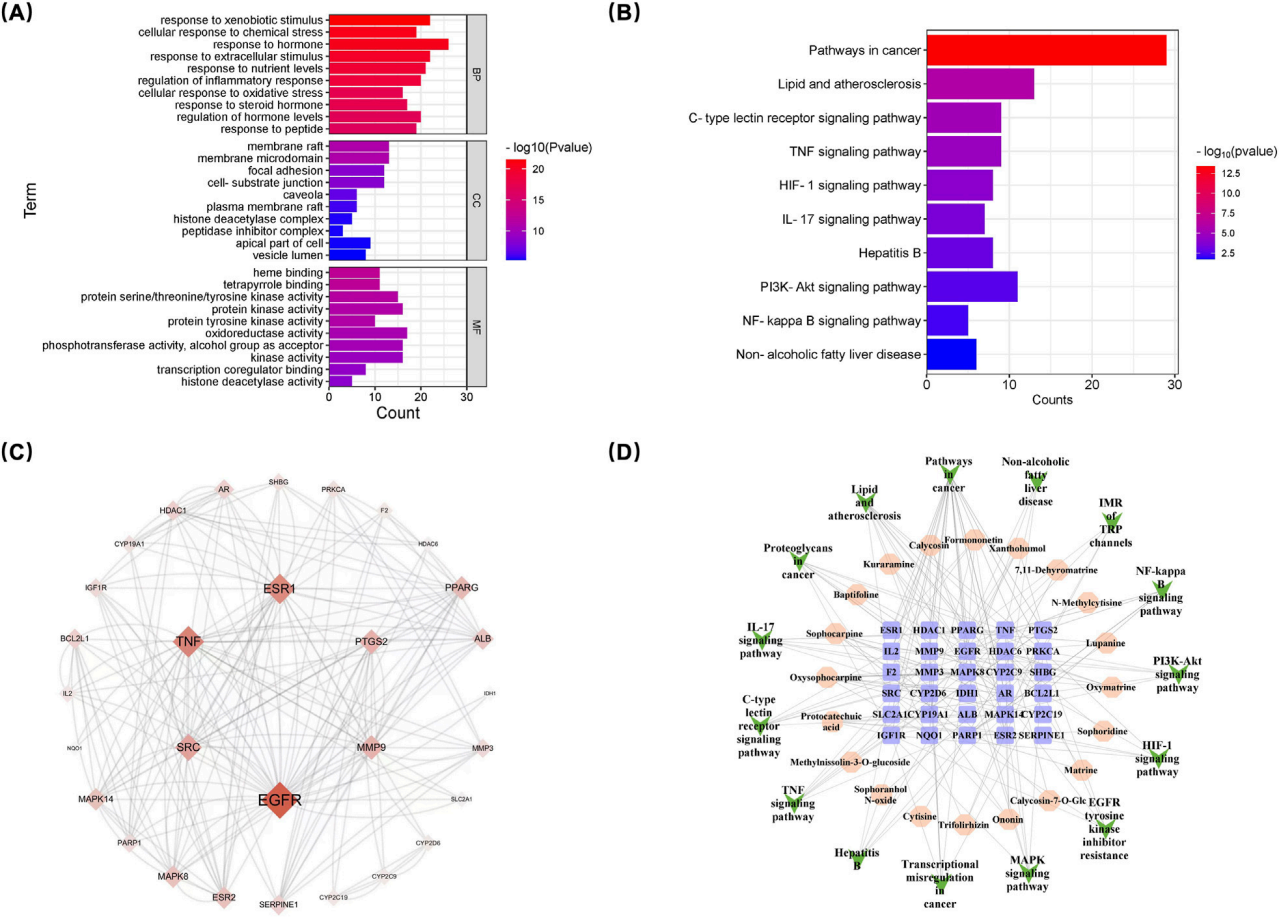
Network pharmacology-based analysis of the anti-HCC mechanisms of the SF-AR herb pair. **(A)** Gene Ontology enrichment analysis of potential targets of absorbed SF-AR constituents. **(B)** KEGG pathway enrichment analysis of potential targets. **(C)** Protein-Protein Interaction network of key potential targets of SF-AR against HCC. **(D)** Constructed “Compound-Target-Pathway” network illustrating the interactions between absorbed SF-AR constituents, their predicted protein targets, and associated anti-HCC pathways.

Functional annotation and enrichment analysis of the 95 primary therapeutic targets of SF-AR herb pair for anti-HCC were performed using the Metascape platform, based on the GO database, along with pathway annotation and enrichment analysis using the KEGG database. Target functional enrichment analysis indicated that the primary therapeutic targets were significantly enriched in biological processes (BP) such as cellular response to chemical stress and regulation of inflammatory response. Furthermore, these targets were associated with molecular functions (MF) including heme binding and protein kinase activity, as well as cellular component (CC) such as focal adhesion (Figure 9A). Pathway analysis identified several significantly enriched pathways, including GnRH signaling pathway, Rap1 signaling pathway, MAPK signaling pathway, PI3K-Akt signaling pathway and Ras signaling pathway, etc. (Figure 9B). The enrichment results underscore the critical involvement of inflammation-related signaling pathways in the therapeutic effects of SF-AR against HCC.

To further identify highly correlated targets, the 95 primary therapeutic targets of SF-AR against HCC were imported into the STRING database to construct a protein-protein interaction (PPI) network. Key targets were selected based on three centrality measures: degree centrality (DC), closeness centrality (CLC), and betweenness centrality (BC). Targets exceeding the median thresholds values (DC > 8, CLC > 0.34, BC > 0.0065) were considered significant, resulting in the identification of 30 key targets, as shown in Figure 9C. In the network, nodes with higher degree values are presented with larger sizes, while deeper colors indicate stronger interactions, emphasizing their significance within the network. The degree values of EGFR, TNF, PTGS2, ESR1, SRC, and MMP9 were among the highest, suggesting that these targets may play critical roles in the molecular mechanisms underlying the anti-HCC effects of SF-AR.

Furthermore, to investigate the potential active constituents of SF-AR, a *compound-target-pathway* network was constructed, as shown in Figure 9D. This network included 20 compounds, 30 key targets, and 15 pathways, highlighting the multi-target intervention characteristics of SF-AR. Ten constituents exhibited degree values exceeding the median threshold, including oxysophocarpine, sophocarpine, calycosin, formononetin, protocatechuic acid, xanthohumol, oxymatrine, ononin, lupinine, and trifolirhizin. These compounds are proposed to be the key bioactive constituents responsible for the anti-HCC effects of SF-AR. The identification of these 10 key bioactive constituents not only provided insights into the pharmacological basis of SF-AR but also emphasizes the complexity of its multi-constituent, multi-target, and multi-pathway intervention mechanisms.

### 3.6 Molecular docking of potential active components and their key targets in anti-HCC

**FIGURE 10:**
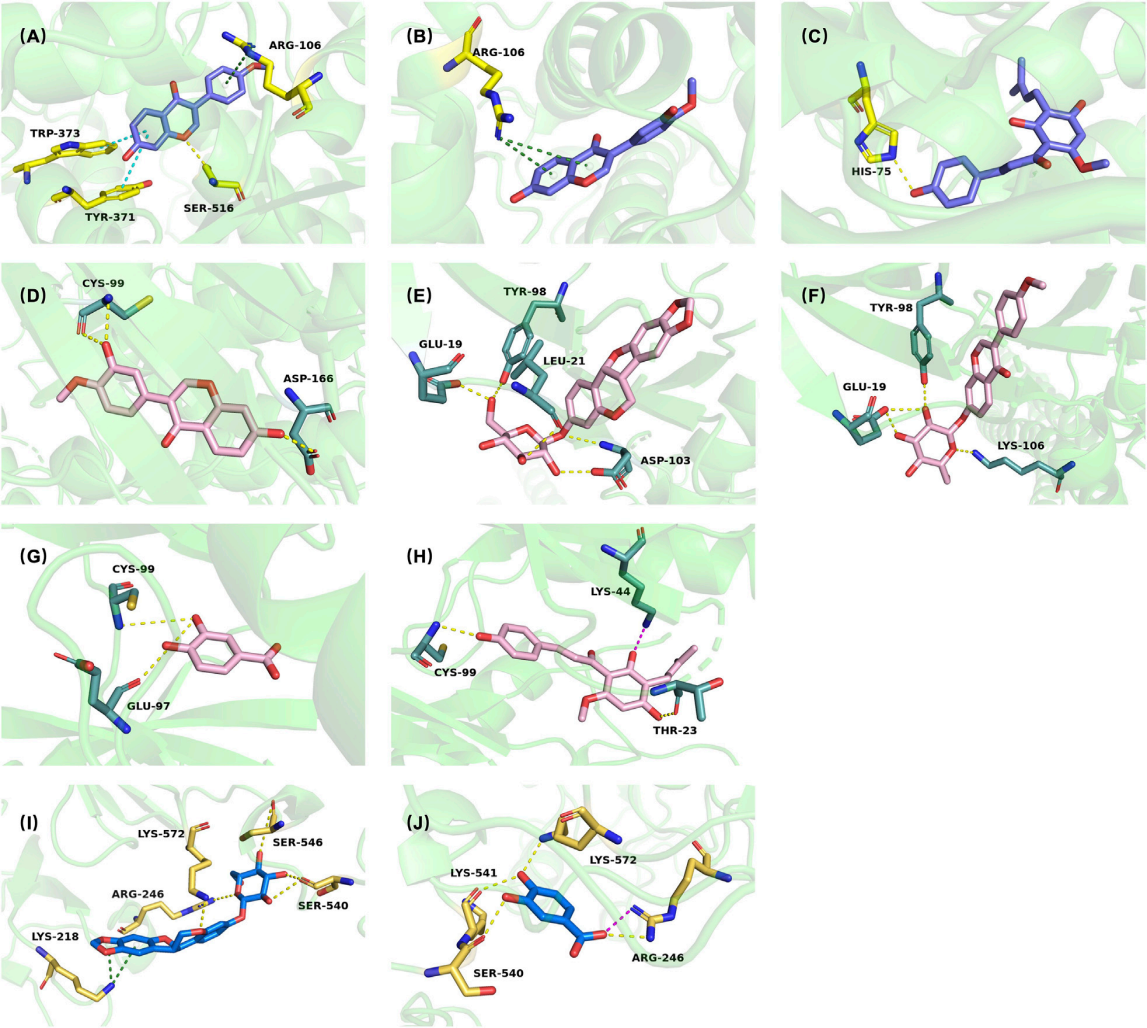
Predicted binding models of key active constituents from SF-AR herb pair with COX2, IKKβ, and NF-κB p65. Predicted binding poses of formononetin **(A)**, calycosin **(B)**, and xanthohumol **(C)** within the active site of COX2. Predicted binding poses of calycosin **(D)**, trifolirhizin **(E)**, ononin **(F)**, protocatechuic acid **(G)**, and xanthohumol **(H)** within the active site of IKKβ. Predicted binding poses of trifolirhizin **(I)** and protocatechuic acid **(J)** within the active site of NF-κB p65.

To investigate the specific interaction of the key active constituents of SF-AR with the key targets in inflammation related pathway, molecular docking was performed. Ten key potential active constituents identified in the *compound-target-pathway* network from the network pharmacology were docked with three key targets, COX2, IKKβ, and NF-κB p65. The detailed docking results are shown in Table 3 and Figure 10.

**TABLE 3:** Molecular docking scores and key binding interactions of selected SF-AR constituents with COX2, IKKβ, and NF-κB p65.

Target	Compound	Docking score	Bonding
COX2	Celecoxib (positive drug)	−11.189	**ARG106**, PHE504, LEU338, ARG499
	Formononetin	−8.879	**ARG106**, TRP373, TYR371, SER516
	Calycosin	−7.908	**ARG106**
	Xanthohumol	−7.466	HID75
IKKβ	TPCA-1 (positive drug)	−7.588	**CYS99**, **ASP103**, **GLU97**
	Calycosin	−9.141	**CYS99**, ASP166
	Trifolirhizin	−8.882	GLU19, LEU21, TYR98, **ASP103**
	Ononin	−8.641	GLU19, TYR98, LYS106
NF-κB p65	Mangiferin (positive drug)	−6.886	**LYS572**, **SER540**, **LYS541**, **ARG246**
	Trifolirhizin	−6.388	ARG236, GLU225, ILE224, GLU222, LYS221
	Protocatechuic acid	−5.92	**ARG246**, **SER540**, **LYS541**, **LYS572**

Using COX2 as the receptor, celecoxib, a selective COX2 inhibitor with known activity, was used as the positive drug. The binding active site of celecoxib with COX2 was considered as the active pocket, and the 10 potential key active constituents of SF-AR were used as ligands for molecular docking. The results showed that the inhibitor celecoxib interacted with amino acid residues such as ARG106, PHE504, LEU338, and ARG499 in COX2. Among the 10 compounds, formononetin, calycosin, and xanthohumol exhibited lower docking energies than the median, indicating stronger binding affinity. Specifically, the phenyl ring of formononetin formed a cation-π bond with ARG106 in COX2’s active center, consistent with the interaction observed with the positive drug celecoxib. Additionally, the phenyl ring of formononetin also formed π-π interactions with TRP373 and TYR371, and the oxygen atom formed a hydrogen bond with SER516 (Figures 10A–C).

For IKKβ, the receptor was based on the structure in PDB ID: 4KIK, with the ligand K-252A bound to IKKβ′s active site. The results indicated that the IKKβ inhibitor TPCA-1 formed multiple hydrogen bonds with amino acid residues such as GLU97, ASP103, and CYS99 in the active pocket. Among the 10 compounds, calycosin, trifolirhizin, ononin, protocatechuic acid, and xanthohumol showed strong binding affinities with IKKβ. Specifically, the hydroxyl group on calycosin formed multiple hydrogen bonds with CYS99 and ASP166 in IKKβ′s active center, similar to the interaction observed with the positive drug (Figures 10D–H).

For NF-κB p65, the protein phosphorylation site of p65 was defined as the active pocket, and p65 inhibitor mangiferin, along with the 10 compounds, were used as ligands for docking. The results revealed that the inhibitor interacted with amino acid residues such as LYS572, SER540, LYS541, and ARG246 in the active pocket through multiple hydrogen bonds. Among the 10 compounds, trifolirhizin and protocatechuic acid from SF exhibited lower docking energies, indicating strong binding affinity with NF-κB p65. Specifically, the hydroxyl group on the saccharide chain of trifolirhizin formed multiple high-energy intermolecular hydrogen bonds with the active center of NF-κB p65, and the phenyl ring formed a cation-π bond with ARG236. However, the two compounds with the best binding energies did not show interaction with the reported phosphorylation site SER468 associated with p65 inhibition (Figures 10I,J).

## 4 Discussion

In this study, the SF-AR herb pair demonstrated significant anti-HCC activity in both cell-based and animal models. SF-AR selectively inhibited the proliferation of HCC cells and induced apoptosis *in vitro* while showing minimal cytotoxicity toward normal hepatic cells. *In vivo*, the combined SF-AR treatment suppressed tumor growth in H22 allograft-bearing mice in a dose-dependent manner. Notably, SF-AR achieved more pronounced tumor suppression than either SF or AR alone, indicating a enhanced anti-tumor effect of the herb pair. This finding supports the traditional rationale of using herb pairs in TCM to enhance therapeutic efficacy through complementary actions.

Chronic inflammation is widely recognized as a key factor in the initiation and progression of HCC, driving alterations in the tumor microenvironment, immune evasion, and sustained cellular proliferation [48]. The involvement of key signaling pathways such as MAPK, PI3K-Akt, and Ras further supports this concept, as these pathways are known to regulate inflammatory responses, cell survival, and proliferation in cancer [49]. Specifically, the MAPK pathway is pivotal in regulating cellular responses to stress, while the PI3K-Akt pathway plays an essential role in promoting cell survival and metabolism under inflammatory conditions [50].

The enrichment of SF-AR’s putative targets in molecular functions such as heme binding and protein kinase activity, as indicated by the GO analysis, also warrants attention. Heme binding is closely related to oxidative stress, a common feature of the inflammatory microenvironment in HCC. Similarly, protein kinase activity is a key regulator of intracellular signaling pathways that govern cell cycle progression and apoptosis [51]. These insights suggest that targeting inflammation-driven signaling axes is central to the herb pair’s anti-HCC mechanism. Further investigations should focus on experimentally validating these pathways to clarify the precise molecular mechanisms by which SF-AR exerts its anti-cancer effects.

The network pharmacology analysis highlighted several hub targets that likely mediate the herb pair’s effects. In the PPI network, proteins such as EGFR, TNF, PTGS2 (COX-2), ESR1, SRC, and MMP9 emerged as highly connected nodes. These are well-known players in HCC pathogenesis. EGFR is a key regulator of cell proliferation and survival, while TNF plays a dual role in promoting inflammation and remodeling of the tumor microenvironment [52]. PTGS2 is widely implicated in inflammation-driven carcinogenesis and is frequently overexpressed in HCC [53]. SRC, a non-receptor tyrosine kinase, governs various oncogenic processes including cell migration, invasion, and angiogenesis. MMP9, a matrix metalloproteinase, is essential for extracellular matrix remodeling and metastasis [54]. The prominence of these targets in the network underscores the relevance of SF-AR’s multi-target approach, as the herb pair appears to engage multiple critical pathways and regulators associated with HCC progression.

The identification of key bioactive constituents through the compound–target–pathway network provides a pharmacological basis for the herb pair’s efficacy. Among the top ten candidates, the alkaloids oxymatrine and matrine, both from SF, have been widely reported for their anti-cancer properties, including induction of apoptosis, inhibition of angiogenesis, and modulation of immune responses [28]. Similarly, the isoflavonoids calycosin and formononetin, which are predominant in AR, are recognized for their anti-inflammatory and anti-proliferative activities, which may synergize with other constituents to enhance the overall therapeutic effect of SF-AR [55]. The inclusion of these compounds among SF-AR’s active constituents not only reinforces the credibility of the network predictions but also aligns with existing literature on their anti-HCC potential. An important observation was the absence of detectable terpenoids, such as astragaloside IV, in our *in vivo* samples despite their presence in the extract. This suggests that these compounds may have poor oral bioavailability or undergo extensive first-pass metabolism. It is also possible that their systemic concentrations were below the detection limit of our analytical method. This underscores the notion that not all chemical constituents present in an herbal extract necessarily achieve systemic exposure in their prototype form to exert direct pharmacological effects. Moving forward, these findings warrant further experimental validation to confirm the predicted interactions between the identified bioactive compounds, their molecular targets, and the associated pathways.

In summary, the molecular docking results provide additional support for the multi-component, multi-target pharmacology of SF-AR. Several representative phytochemicals–most prominently formononetin, calycosin, xanthohumol, trifolirhizin, and protocatechuic acid–exhibited binding affinities and interaction patterns comparable to those of the known inhibitors celecoxib, TPCA-1, and mangiferin when docked to COX2, IKKβ, and NF-κB p65, respectively. The key ligand–target contacts observed in these docking models, such as cation–π stacking with ARG106 in COX2, hydrogen-bond networks with CYS99/ASP166 in IKKβ, and dual hydrogen-bond plus cation–π interactions with ARG236 in p65, mirrored the binding modes of the positive drugs, lending credibility to the predicted interactions.

Particularly noteworthy are the docking results for NF-κB p65. While representative compounds showed strong predicted binding affinities, their interaction sites did not directly involve the key phosphorylation residue, SER468. This finding suggests two possibilities: the compounds may act through an allosteric modulatory mechanism by binding to a different site and inducing inhibitory conformational changes, or this result may reflect the inherent limitations of static docking models that do not fully capture protein dynamics. Therefore, while our results support a possible direct interaction with NF-κB p65, further experimental studies are required to elucidate the precise binding site and inhibitory mechanism.

These findings collectively reinforce the concept that SF-AR exerts its anti-HCC activity through the concerted engagement of multiple bioactive constituents with several nodes of the inflammatory cascade, rather than relying on a single dominant compound–target pair. The demonstrated multi-ligand, multi-target binding profile offers a molecular rationale for the observed enhanced efficacy of the SF-AR herb pair. In essence, the integrative approach of combining phytochemical profiling, *in vivo* absorption studies, network pharmacology, and molecular docking has established a link between the herb pair’s well-defined chemical composition and its anti-HCC effects. Importantly, by focusing the network pharmacology analysis only on constituents confirmed to be absorbed *in vivo*, this study provides a more pharmacologically relevant basis for the herb pair’s mechanism of action than a simple chemical inventory. This comprehensive understanding lays a foundation for further development of SF-AR as a safe, multitarget therapeutic candidate for HCC, and it underscores the value of traditional herb pairs as multi-component interventions in modern oncology research.

## 5 Conclusion

This study successfully demonstrated the anti-HCC efficacy of the SF-AR herb pair. The SF-AR extract inhibited HepG2 cell proliferation, promoted apoptosis, and reduced H22 tumor allograft growth in mice in a dose-dependent manner, without overt toxicity. A primary achievement of this work is the comprehensive phytochemical characterization of the SF-AR extract using LC-QTOF-MS. This analysis led to the delineation of 95 constituents, including 37 flavonoids, 23 alkaloids, 12 terpenoids, alongside organic acids, amino acids, nucleic acids and others. Crucially, detailed collision-induced dissociation pathways were summarized for key compound classes, such as RDA cleavages for isoflavonoids and the identification of diagnostic ions for matrine-type alkaloids, which aided in navigating the structural complexity of the extract. Furthermore, investigation into the *in vivo* fate of these compounds revealed that 22 prototype constituents, primarily alkaloids and flavonoids, were absorbed into rat plasma and subsequently distributed to the liver, kidney, and spleen, confirming their systemic exposure and bioavailability.

The identification of these absorbed phytochemicals provided a concrete basis for predicting the mechanisms underlying SF-AR’s anti-HCC action. Network pharmacology analysis associated these systemically available constituents with established HCC-related targets, implicating inflammation- and cell survival-related signaling pathways in the herb pair’s therapeutic effects. Molecular docking studies further supported these predictions by demonstrating favorable binding interactions between representative identified compounds and key proteins within these pathways. Collectively, these findings establish a critical link between the observed anti-HCC effects of the SF-AR herb pair and its well-defined, multi-class phytochemical profile. This detailed chemical understanding, coupled with preliminary mechanistic insights, provides a solid foundation for the continued investigation and development of SF-AR as a potentially safe and effective multi-target therapeutic candidate for HCC treatment. Future work should aim to experimentally validate our *in silico* predictions. Key absorbed constituents should be investigated for their direct effects on predicted targets to confirm the precise mechanisms of action for the SF-AR herb pair and to further clarify the enhanced interactions of the identified bioactive compounds.

## Data Availability

The original contributions presented in the study are publicly available. This data can be found here: 10.6084/m9.figshare.30447968.
