# Supplementary material for: Elucidating the phytochemical profile of Sophorae Flavescentis Radix-Astragali Radix herb pair: an integrated LC-QTOF-MS/MS, pharmacological activity, and network pharmacology study on anti-hepatocellular carcinoma effects
Source: Front Chem. 2025 Nov 7;13:1687098. doi: 10.3389/fchem.2025.1687098 (PMC12634525; doi:10.3389/fchem.2025.1687098)
Supplement: Supplementary file 1 [file Table1.docx]

Supplementary Material

# Supplementary Tables

**Supplementary Table 1.** Reference standards used for the identification and characterization of constituents in the SF-AR herb pair.

| NO. | Name | Manufacturer |
| --- | --- | --- |
| 1 | Calycosin-7-O-Glc | Chengdu Chroma Biotechnology Ltd. |
| 2 | Calycosin | Chengdu Chroma Biotechnology Ltd. |
| 3 | Ononin | Chengdu Chroma Biotechnology Ltd. |
| 4 | Trifolirhizin | Chengdu Chroma Biotechnology Ltd. |
| 5 | Formononetin | Chengdu Chroma Biotechnology Ltd. |
| 6 | Kurarinon | Chengdu Chroma Biotechnology Ltd. |
| 7 | Oxymatrine | Chengdu Chroma Biotechnology Ltd. |
| 8 | Oxysophocarpine | Chengdu Chroma Biotechnology Ltd. |
| 9 | Sophoridine | National Institutes for Food and Drug control |
| 10 | N-methyI cytisine | Chengdu Chroma Biotechnology Ltd. |
| 11 | Matrine | Chengdu Chroma Biotechnology Ltd. |
| 12 | Sophocarpine | Chengdu Chroma Biotechnology Ltd. |
| 13 | Astragaloside Ⅳ | National Institutes for Food and Drug control |
| 14 | Astragaloside II | Chengdu Chroma Biotechnology Ltd. |

**Supplementary Table 2.** Phytochemicals identified in the SF-AR herb pair by LC-QTOF-MS.

| NO | t_R_ (min) | Compound | Formula | Adduct | Found (*m/z*) | Error (ppm) | Fragment ions | SF | AR | SF-AR |
| --- | --- | --- | --- | --- | --- | --- | --- | --- | --- | --- |
| 1 | 12.5 | Rhamnocitrin-Hex | C_22_H_22_O_11_ | [M-H]^-^ | 461.1087 | -0.5 | 299.0576, 284.0325, 267.0391, 227.0368, 212.0465, 175.0886, 59.0179 | √ | √ | √ |
| 2 | 18.42 | Mirificin | C_26_H_28_O_13_ | [M+H]^+^ | 549.1591 | -2.1 | 255.0649, 417.1210,199.0721, 137.0247 | ND | ND | √ |
|  |  |  |  | [M-H]^-^ | 547.1446 | -2.1 | 281.0426, 253.0500, 119.9501 |  |  |  |
| 3 | 19.46 | Iso-mirificin | C_26_H_28_O_13_ | [M+H]^+^ | 549.159 | -2.2 | 255.0651, 417.1313, 249.1952, 137.0231 | ND | ND | √ |
|  |  |  |  | [M-H]^-^ | 547.1452 | -1 | 281.0426, 253.0500, 119.9501 |  |  |  |
| 4 | 20.05 | Daidzin | C_21_H_20_O_9_ | [M+H]^+^ | 417.1175 | -1.1 | 137.0296, 199.0808, 227.0673, 255.0654 | ND | √ | √ |
|  |  |  |  | [M-H]^-^ | 415.1036 | 0.4 | (-) |  |  |  |
| 5 | 20.57 | Calycosin-7-O-Glc-6''-O-malonate | C_25_H_24_O_13_ | [M+H]^+^ | 533.1279 | -2 | 285.0761, 270.0524, 253.0495, 225.0558, 213.0533, 197.0595, 137.0229 | ND | √ | √ |
|  |  |  |  | [M-H]^-^ | 531.1134 | -1.8 | 283.0599, 268.0379, 240.0406, 211.0391, 148.0130 |  |  |  |
| 6 | 22.69 | Isomer calycosin-7-O-Glc-6''-O-Mal | C_25_H_24_O_13_ | [M+H]^+^ | 533.1276 | -2.5 | 253.0465, 270.0518, 285.0749 | √ | √ | √ |
| 7* | 23.76 | Calycosin-7-O-Glc | C_22_H_22_O_10_ | [M+H]^+^ | 447.1278 | -1.7 | 285.0748, 270.0520, 225.0544, 137.0229 | √ | √ | √ |
|  |  |  |  | [M-H]^-^ | 445.1128 | -2.7 | 283.0609, 268.0370, 240.0403, 211.0404, 174.9913, 150.9503 |  |  |  |
| 8 | 25.99 | Rhamnocitrin 3,4'-di-O-Glc | C_28_H_32_O_16_ | [M-H]^-^ | 623.1598 | -3.1 | 563.1359, 269.0440 | √ | √ | √ |
| 9 | 26.19 | 10-dihydroxy-9-methoxypterocarpan-3-O-Glc-6'-O-Mal | C_25_H_26_O_13_ | [M+H]^+^ | 535.1459 | 2.4 | (-) | ND | √ | √ |
| 10 | 27.92 | Apigenin-Hex | C_21_H_20_O_10_ | [M+H]^+^ | 433.1123 | -1.4 | 271.0598, 153.0155, 215.0689, 253.0451, 270.2183 | √ | ND | √ |
|  |  |  |  | [M-H]^-^ | 431.0979 | -1.2 | 269.0446, 239.0485, 211.0407, 134.9460, 59.0203 |  |  |  |
| 11 | 28.73 | Odoratin-7-O-Glc-6''-O-Mal | C_26_H_26_O_14_ | [M+H]^+^ | 563.1392 | -0.6 | 563.1384, 300.0682, 315.086 | ND | √ | √ |
| 12 | 30.65 | 6,4'-dimethoxyisoflavone-7-O-Glc | C_23_H_24_O_10_ | [M+H]^+^ | 461.1435 | -1.5 | 162.0214, 238.0644, 243.0964, 266.0594, 299.0902 | ND | √ | √ |
| 13 | 31.6 | Formononetin-7-O-Glc-6''-O-malonate | C_25_H_24_O_12_ | [M+H]^+^ | 517.1329 | -2.3 | 269.0807, 254.0581, 213.0904 | √ | √ | √ |
|  |  |  |  | [M-H]^-^ | 515.1175 | -3.8 | 309.0742, 267.0659, 252.0425, 174.9899 |  |  |  |
| 14 | 32.21 | 3-Hydro-9-methoxypterocarpan- hexoside | C_22_H_24_O_10_ | [M+H]^+^ | 449.1422 | -4.4 | 287.0902, 255.0633, 227.0687, 177.0518, 153.0534 | √ | √ | √ |
|  |  |  |  | [M-H]^-^ | 447.1283 | -1.3 | (-) |  |  |  |
| 15 | 32.38 | Pratensein-7-O-Glc | C_22_H_22_O_11_ | [M+H]^+^ | 463.1227 | -1.7 | 153.0158, 241.0490, 269.0458, 286.0492, 301.0703 | ND | √ | √ |
|  |  |  |  | [M-H]^-^ | 461.1098 | 1.9 | (-) |  |  |  |
| 16 | 32.45 | Biochanin A | C_16_H_12_O_5_ | [M-H]^-^ | 283.061 | -0.7 | 268.0366, 239.0337, 211.0400, 195.0458, 148.0172, 91.0207 | √ | √ | √ |
| 17 | 33.19 | Daidzein | C_15_H_10_O_4_ | [M+H]^+^ | 255.0654 | 0.7 | 137.0209, 145.0248, 153.0694, 168.0584, 199.0783, 227.0633 | √ | ND | √ |
|  |  |  |  | [M-H]^-^ | 253.0502 | -1.5 | (-) |  |  |  |
| 18 | 34.36 | Kurarinol O | C27H30O13 | [M-H]^-^ | 561.1595 | -3.4 | 267.0655, 252.0431, 223.0427, 195.0437 | √ | ND | √ |
| 19* | 38.23 | Calycosin | C_16_H_12_O_5_ | [M+H]^+^ | 285.0757 | -0.1 | 137.0229, 157.0639, 197.0588, 213.0538, 214.0614, 225.0528, 242.0574, 253.0485, 270.0515 | √ | √ | √ |
|  |  |  |  | [M-H]^-^ | 283.0613 | 0.4 | 254.0575, 211.0363, 185.0601, 183.0792, 133.0300, 89.0447, 59.0167 |  |  |  |
| 20 | 39.54 | Kenusanone I | C_21_H_22_O_6_ | [M+H]^+^ | 370.1416 | -2.6 | (-) | √ | ND | √ |
|  |  |  |  | [M-H]^-^ | 369.1346 | 0.7 | 341.1398, 247.0647, 207.1024, 192.0772, 137.0271, 124.0165 |  |  |  |
| 21* | 39.82 | Ononin | C_22_H_22_O_9_ | [M+H]^+^ | 431.134 | -1.3 | 269.0798, 254.0570, 213.0908 | √ | √ | √ |
|  |  |  |  | [M+AcO-H]^-^ | 489.1383 | -4 | 267.0657, 252.0423, 223.0397, 59.0174 |  |  |  |
| 22 | 41 | Sophoraflavanone B | C_20_H_20_O_5_ | [M-H]^-^ | 339.1237 | -0.3 | 263.0744, 309.0709, 324.0962 | √ | ND | √ |
| 23 | 42.49 | 7-O-methyl-vesticarpan | C_17_H_16_O_5_ | [M+H]^+^ | 301.1069 | -0.4 | 78.0476, 106.0410, 152.0467, 167.0699, 181.0639, 225.0546, 269.0791 | ND | √ | √ |
| 24 | 42.48 | Methylnissolin-3-O-glucoside | C_23_H_26_O_10_ | [M+H]^+^ | 463.159 | -1.9 | 134.0364, 152.0454, 167.0693, 191.0681, 301.1052 | ND | √ | √ |
|  |  |  |  | [M-H]^-^ | 461.1437 | -3.6 | 299.0545, 267.0639, 252.0435, 121.0311, 59.0166 |  |  |  |
| 25 | 43.17 | Mucronulatol | C_17_H_18_O_5_ | [M+H]^+^ | 303.1226 | -0.4 | 77.0457, 133.068, 161.0581, 167.0715 | ND | √ | √ |
| 26* | 43.34 | Trifolirhizin | C_22_H_22_O_10_ | [M+H]^+^ | 447.1282 | -0.8 | 147.0438, 151.0392, 175.0389, 255.0648, 285.0762 | √ | ND | √ |
|  |  |  |  | [M-H]^-^ | 445.1126 | -3.2 | 283.0604, 255.0650, 240.0430, 211.0748, 137.0247, 121.0306 |  |  |  |
| 27 | 45.78 | Formononetin-7-O-Glc-6"-O-acetate | C_24_H_24_O_10_ | [M+H]^+^ | 473.1432 | -2.1 | 213.0888, 254.0534, 269.0781 | √ | √ | √ |
| 28 | 46.2 | Vesticarpan (3,10-dihydroxy-9- methoxypterocarpan) | C_16_H_14_O_5_ | [M+H]^+^ | 287.0901 | -4.5 | 110.0376, 138.0266, 153.0526, 255.0655 | ND | √ | √ |
|  |  |  |  | [M-H]^-^ | 285.0759 | -3.3 | (-) |  |  |  |
| 29 | 46.75 | Afrormosin | C_17_H_14_O_5_ | [M+H]^+^ | 299.0919 | 1.6 | 111.0449, 132.0592, 168.0560, 227.0662, 256.0703, 284.0682 | ND | √ | √ |
| 30 | 47.32 | Sophoraisoflavanone A | C_21_H_22_O_6_ | [M+H]^+^ | 371.149 | -2.7 | 129.0503, 151.0395, 179.0337, 235.0963, 254.0578, 297.0754, 315.0853 | √ | √ | √ |
|  |  |  |  | [M-H]^-^ | 369.1334 | -2.6 | 341.1373, 207.1028, 138.0329 |  |  |  |
| 31* | 48.08 | Formononetin | C_16_H_12_O_4_ | [M+H]^+^ | 269.0808 | 0 | 137.0226, 152.0604, 169.0633, 181.0643, 197.0585, 213.0895, 225.0531, 237.0541, 253.0490 | √ | √ | √ |
| 32 | 49.2 | Xanthohumol | C_21_H_22_O_5_ | [M+H]^+^ | 355.1537 | -0.8 | 113.0231, 151.0381, 179.0339, 235.0960, 299.0930 | √ | ND | √ |
|  |  |  |  | [M-H]^-^ | 353.139 | -1.3 | 233.0807, 175.0062, 119.0512 |  |  |  |
| 33 | 50.29 | Methylnissolin | C_17_H_16_O_5_ | [M+H]^+^ | 301.1072 | 0.7 | 167.0699, 152.0459, 147.0437, 134.036 | ND | √ | √ |
|  |  |  |  | [M-H]^-^ | 299.0927 | 0.5 | 284.0689, 269.0444, 241.0504, 223.0281, 213.0573, 171.0460, 133.0309, 59.0199 |  |  |  |
| 34 | 50.6 | Isomucronulatol | C_17_H_18_O_5_ | [M+H]^+^ | 303.1219 | -2.7 | 118.0414, 123.0440, 133.0638, 152.0460, 161.0583, 167.0700, 193.0791, 259.2821 | ND | √ | √ |
|  |  |  |  | [M-H]^-^ | 301.1077 | -1.4 | 286.0840, 271.0613, 256.0379, 241.2173, 225.0273, 164.0485, 149.0254, 135.0468, 121.0294, 109.0312, 93.0407 |  |  |  |
| 35 | 50.81 | Kurarinol I | C_26_H_30_O_7_ | [M-H]^-^ | 453.1901 | -3.8 | 275.1643, 177.0192, 149.0252, 139.0410, 121.0312, 105.0351 | √ | ND | √ |
| 36 | 51.26 | Maackiain | C_16_H_12_O_5_ | [M+H]^+^ | 285.076 | -0.8 | 93.0358, 123.0444, 151.0388, 175.0407, 255.0651 | √ | ND | √ |
|  |  |  |  | [M-H]^-^ | 283.0611 | -0.4 | 268.0375, 254.0579, 240.0429, 211.0404, 185.0617, 167.0507, 133.0309 |  |  |  |
| 37* | 51.9 | Kurarinon | C_26_H_30_O_6_ | [M+H]^+^ | 439.2033 | -2.3 | 149.0495, 179.0314, 254.0545, 297.0739, 303.1644, 315.0813 | √ | ND | √ |
|  |  |  |  | [M-H]^-^ | 437.1954 | -3.6 | 275.1645, 161.0247, 151.0405, 117.0356, 78.9619 |  |  |  |
| 38 | 5.7 | Lupinine | C_10_H_19_NO | [M+H]^+^ | 170.1537 | -1.7 | 152.1437, 136.1120, 124.1114, 110.0969, 96.0812, 84.0836 | √ | ND | √ |
| 39 | 7.3 | 5α,9α-dehydromatrine | C_15_H_24_N_2_O_3_ | [M+H]^+^ | 281.1861 | 0.4 | 150.1262, 162.0899, 221.1280, 245.1636, 263.1747 | √ | ND | √ |
|  |  |  |  | [M-H]^-^ | 279.171 | -1.4 | (-) |  |  |  |
| 40 | 8.3 | Sophoranhol N-oxide | C_15_H_24_N_2_O_3_ | [M+H]^+^ | 281.1859 | -0.2 | 134.0972, 136.1117, 148.111, 150.1272, 193.1679, 218.1811, 221.1279, 222.184, 263.1757, 264.1824 | √ | ND | √ |
|  |  |  |  | [M-H]^-^ | 279.1701 | -4.6 | 161.0266, 59.0171 |  |  |  |
| 41 | 8.38 | 7a-Hydroxysophocarpine | C_15_H_22_N_2_O_3_ | [M+H]^+^ | 279.1701 | -0.9 | 122.0597, 150.1269, 177.1378, 243.1505, 261.1599 | √ | ND | √ |
|  |  |  |  | [M-H]^-^ | 277.1545 | -4.5 | (-) |  |  |  |
| 42 | 8.78 | Sophoranol | C_15_H_24_N_2_O_2_ | [M+H]^+^ | 265.1908 | -0.8 | 136.1111, 150.1265, 162.1263, 177.1372, 205.1321, 247.1786 | √ | ND | √ |
| 43 | 9.27 | 7α-Hydroxysophoramine | C_15_H_20_N_2_O_2_ | [M+H]^+^ | 261.1597 | -0.3 | 136.1116, 148.1004, 215.1168, 243.1477 | √ | ND | √ |
| 44 | 9.56 | 9α-Hydroxysophoramine | C_15_H_20_N_2_O_2_ | [M+H]^+^ | 261.1596 | -0.4 | 150.1267, 177.1373, 243.1482, 122.0957 | √ | ND | √ |
| 45 | 10.47 | Cytisine | C_11_H_14_N_2_O | [M+H]^+^ | 191.1177 | -0.9 | 80.0513, 91.0563, 118.0646, 133.0523, 148.0750, 174.0907 | √ | ND | √ |
| 46* | 11.96 | Oxymatrine | C_15_H_24_N_2_O_2_ | [M+H]^+^ | 265.1908 | -1 | 98.0963, 136.1100, 148.1105, 150.1260, 161.0827, 176.1066, 205.1321, 247.1787 | √ | ND | √ |
| 47* | 13.11 | Oxysophocarpine | C_15_H_22_N_2_O_2_ | [M+H]^+^ | 263.1751 | -1 | 98.0975, 136.1113, 150.1260, 177.1372, 203.1162, 245.1621 | √ | ND | √ |
|  |  |  |  | [M-H]^-^ | 261.16 | -3.3 | 259.1474, 243.1592, 163.1251, 59.0188 |  |  |  |
| 48 | 13.13 | Kuraramine | C_12_H_18_N_2_O_2_ | [M+H]^+^ | 223.1439 | -0.9 | 114.0897, 122.0600, 132.0433, 147.0658, 162.0897, 191.1150, 203.1152, 221.1254 | √ | ND | √ |
|  |  |  |  | [M-H]^-^ | 221.1297 | 0.7 | 191.1201, 148.0779, 120.0469, 94.0321 |  |  |  |
| 49 | 14.38 | 5α,9α-isodehydromatrine | C_15_H_24_N_2_O_3_ | [M+H]^+^ | 281.1859 | -0.2 | 83.0740, 110.0962, 124.1114, 138.1269, 150.1269, 222.1351, 263.1740 | √ | ND | √ |
| 50* | 15.51 | Sophoridine | C_15_H_24_N_2_O | [M+H]^+^ | 249.196 | -0.7 | 84.0819, 152.1427, 249.1951, 136.1117, 148.1108, 150.1265, 176.1066 | √ | ND | √ |
| 51 | 15.63 | Isokuraramine | C_12_H_18_N_2_O_2_ | [M+H]^+^ | 223.1441 | -0.2 | 84.0809, 104.0497, 114.0911, 132.0449, 147.0679, 162.0935, 191.1163, 203.1148, 221.1264 | √ | ND | √ |
| 52 | 16.89 | Baptifoline | C_15_H_20_N_2_O_2_ | [M+H]^+^ | 261.1597 | -0.2 | 70.0677, 94.0655, 96.0820, 114.0920, 146.0954, 164.1067, 243.1494 | √ | ND | √ |
| 53* | 18.26 | N-methyI cytisine | C_12_H_16_N_2_O | [M+H]^+^ | 205.1334 | -0.7 | 146.0604, 58.0681 | √ | ND | √ |
| 54 | 18.88 | Mamanine | C_15_H_22_N_2_O_2_ | [M+H]^+^ | 263.1753 | -0.4 | 70.0683, 84.0825, 98.0975, 122.0603, 154.1227, 160.0748, 203.1175, 231.1492, 245.1653, 261.1601 | √ | ND | √ |
| 55 | 19.65 | Lupanine | C_15_H_24_N_2_O | [M+H]^+^ | 249.196 | -0.5 | 84.0820, 136.1118, 166.1221, 206.1517, 231.1846, 249.1953 | √ | ND | √ |
| 56 | 21.62 | Isolupanine | C_15_H_24_N_2_O | [M+H]^+^ | 249.1959 | -1 | 98.0976, 114.0915, 122.0966, 136.1121, 148.1116, 150.1272, | √ | ND | √ |
|  |  |  |  |  |  |  | 166.1212, 231.1861, 249.1965 |  |  |  |
| 57 | 23.57 | 7,11-Dehyromatrine | C_15_H_22_N_2_O | [M+H]^+^ | 247.1804 | -0.3 | 91.0553, 120.0807, 150.1271, 148.1111, 176.1059, 188.1419, 229.1691 | √ | ND | √ |
| 58* | 28.84 | Matrine | C_15_H_24_N_2_O | [M+H]^+^ | 249.196 | -0.7 | 98.0964, 136.1121, 138.12780, 148.1109, 150.1272, 176.1446, 204.1379, 231.1840, 247.1793 | √ | ND | √ |
| 59 | 30.1 | Anagyrine | C_15_H_20_N_2_O | [M+H]^+^ | 245.165 | 0.8 | 98.0974, 148.1109 | √ | ND | √ |
| 60* | 32.17 | Sophocarpine | C_15_H_22_N_2_O | [M+H]^+^ | 247.1805 | -0.1 | 108.0814, 136.1121, 150.1299, 179.1533, 245.1666 | √ | ND | √ |
| 61 | 39.92 | Cycloastragenol | C_30_H_50_O_5_ | [M+H]^+^ | 491.3729 | -0.4 | 114.0918, 228.1628, 286.0761, 341.2429, 435.3414, 455.3303 | ND | √ | √ |
| 62 | 43.83 | AstragalosideⅤ | C_47_H_78_O_19_ | [M+H]^+^ | 947.5191 | -2 | 125.0966, 143.1055, 305.1624, 419.3280, 437.3393, 455.3570, 455.3356, 473.3609, 587.3945, 617.3977, 767.4797 | ND | √ | √ |
|  |  |  |  | [M-H]^-^ | 945.5059 | -0.5 | 783.4703 |  |  |  |
| 63 | 45.56 | Soyasaponin I | C_48_H_78_O_18_ | [M+H]^+^ | 943.5239 | -2.3 | 221.1960, 305.0863,405.3532, 423.3611, 441.3739, 599.3944, 617.3956, 781.4714,797.4638 | √ | √ | √ |
|  |  |  |  | [M-H]^-^ | 941.5084 | -3.4 | 784.4712, 715.1330, 616.3991, 247.0718 |  |  |  |
| 64 | 46.29 | Ziyuglycoside I | C_41_H_66_O_13_ | [M+H]^+^ | 767.4552 | -3.2 | 143.1079, 185.1265, 251.1755, 279.2096, 329.2458, 419.3377, 425.3403, 437.3383, 455.3553 | ND | √ | √ |
|  |  |  |  | [M-H]^-^ | 765.4411 | -2.6 | (-) |  |  |  |
| 65* | 46.3 | Astragaloside Ⅳ | C_41_H_68_O_14_ | [M+H]^+^ | 785.4669 | -1.6 | 143.1059, 297.2216, 401.3138, 419.3310, 437.3422, 455.35, 473.3618, 569.4009, 587.3947 | ND | √ | √ |
|  |  |  |  | [M+AcO-H]^-^ | 843.4741 | -0.8 | 783.4489, 651.4125, 621.3810, 489.3578, 179.0564, 119.0346 |  |  |  |
| 66 | 46.63 | Astragaloside Ⅲ | C_41_H_68_O_14_ | [M+H]+ | 785.4682 | -2.2 | 785.4245, 437.3283, 199.1487, 143.1105 | ND | √ | √ |
|  |  |  |  | [M+AcO-H]^-^ | 843.4725 | -2.7 | 783.4489, 489.3600, 161.0454 |  |  |  |
| 67* | 47.86 | Astragaloside II | C_43_H_70_O_15_ | [M+H]^+^ | 827.4771 | -2 | 157.0493, 175.0589, 315.2362, 437.3386, 455.3494, 473.3604, | ND | √ | √ |
|  |  |  |  |  |  |  | 593.3565, 611.3970, 647.4126 |  |  |  |
|  |  |  |  | [M+AcO-H]^-^ | 885.4824 | -3.3 | 825.4586, 765.4387, 603.3672, 363.2482, 179.0553 |  |  |  |
| 68 | 48.01 | Cycloastragenol-6-O-β-D-glucoside | C_36_H_60_O_10_ | [M+H]^+^ | 653.4259 | -2.4 | 455.3482, 419.3195, 321.2535, 277.2109, 143.1053, 125.0965 | ND | √ | √ |
|  |  |  |  | [M+FA-H]^-^ | 699.4323 | -0.4 | (-) |  |  |  |
| 69 | 48.74 | Isoastragaloside II | C_43_H_70_O_15_ | [M+H]^+^ | 827.4769 | -2.3 | 123.1139, 125.0956, 141.1251, 143.1059, 175.0597, 297.2212, 401.3200, 437.3435, 455.3545, 473.3739, 611.3830 | ND | √ | √ |
|  |  |  |  | [M+AcO-H]^-^ | 885.4824 | -3.3 | 825.4586, 765.4387, 603.3672, 363.2482, 179.0553 |  |  |  |
| 70 | 49.3 | Cyclocephaloside II | C_43_H_70_O_15_ | [M+H]^+^ | 827.4765 | -2.7 | 143.1067, 175.0587, 297.2204, 407.3278, 419.3266, 437.3407, | ND | √ | √ |
|  |  |  |  |  |  |  | 455.3482, 455.3729, 593.3689, 611.4098 |  |  |  |
|  |  |  |  | [M+AcO-H]^-^ | 885.4849 | -0.5 | 826.4640, 765.4521, 646.4200, 363.1942, 303.2331 |  |  |  |
| 71 | 50.83 | Astragaloside I | C_45_H_72_O_16_ | [M+H]^+^ | 869.4875 | -2.1 | 143.1053, 157.0483, 217.0684, 437.3431, 653.4219, 671.4124, 815.4694 | ND | √ | √ |
|  |  |  |  | [M-H]^-^ | 867.471 | -4.3 | (-) |  |  |  |
| 72 | 51.56 | Isoastragaloside I | C_45_H_72_O_16_ | [M+H]^+^ | 869.4873 | -2.3 | 143.1061, 157.0500, 217.0687, 437.3364, 635.4001, 671.4112, 851.4651 | ND | √ | √ |
|  |  |  |  | [M-H]^-^ | 867.471 | -4.3 | (-) |  |  |  |
| 73 | 2.04 | Citric acid | C_6_H_8_O_7_ | [M+H]^+^ | 193.0343 | -0.1 | 68.9990, 83.0122, 101.0412, 111.0060, 129.0193, 138.9992 | √ | √ | √ |
|  |  |  |  | [M-H]^-^ | 191.0203 | 3 | 87.0110, 67.0224, 57.0389 |  |  |  |
| 74 | 2.29 | Glutamic acid | C_5_H_9_NO_4_ | [M+H]^+^ | 148.06 | -2.6 | 56.0529, 84.0452, 102.0558 | √ | √ | √ |
| 75 | 2.33 | Hexose | C_6_H_12_O_6_ | [M-H]^-^ | 179.0548 | 2 | 59.0181, 71.0171, 89.0258, 101.0264 | √ | √ | √ |
| 76 | 2.44 | Aspartic acid | C_4_H_8_N_2_O_3_ | [M+H]^+^ | 133.0605 | -2.1 | 70.0657, 74.0238, 87.0536, 116.0345 | √ | √ | √ |
| 77 | 2.44 | Arginine | C_6_H_14_N_4_O_2_ | [M+H]^+^ | 175.1186 | -2.2 | (-) | √ | √ | √ |
|  |  |  |  | [M-H]^-^ | 173.1048 | 2 | 131.0840, 113.0740, 83.0638 |  |  |  |
| 78 | 2.48 | Melitriose | C_18_H_32_O_16_ | [M-H]^-^ | 503.1604 | -2.7 | (-) | √ | √ | √ |
| 79 | 2.49 | Stachyose | C_24_H_42_O_21_ | [M+H]^+^ | 667.228 | -1.7 | (-) | √ | √ | √ |
|  |  |  |  | [M-H]^-^ | 665.2136 | -1.5 | (-) |  |  |  |
| 80 | 2.57 | Histidine | C_6_H_9_N_3_O_2_ | [M+H]^+^ | 156.0763 | -3 | (-) | √ | √ | √ |
|  |  |  |  | [M-H]^-^ | 154.0629 | 4.4 | (-) |  |  |  |
| 81 | 2.61 | Proline | C_5_H_9_NO_2_ | [M+H]^+^ | 116.0705 | -1 | 70.0664 | √ | √ | √ |
|  |  |  |  | [M-H]^-^ | 114.0565 | 4.1 | (-) |  |  |  |
| 82 | 2.66 | Sucrose | C_12_H_22_O_11_ | [M-H]^-^ | 341.1081 | -2.3 | 59.0180, 89.0264, 119.0362, 179.0567 | √ | √ | √ |
| 83 | 2.74 | Choline | C_5_H_13_NO | [M+H]^+^ | 104.1069 | -0.4 | 58.0676, 60.0827 | √ | √ | √ |
| 84 | 2.85 | Azelaic acid | C_9_H_16_O_4_ | [M-H]^-^ | 187.0979 | 1.9 | 97.0688, 125.0983, 143.1116, 169.0906 | √ | √ | √ |
| 85 | 4.05 | Sinapic acid | C_11_H_12_O_5_ | [M-H]^-^ | 223.0615 | 1.4 | 91.0564, 117.0724 | ND | √ | √ |
| 86 | 4.06 | Tyrosine | C_9_H_11_NO_3_ | [M+H]^+^ | 182.0809 | -1.7 | 77.0398, 91.0547, 136.0744, 165.0514 | √ | √ | √ |
| 87 | 4.09 | Leucine | C_6_H_13_NO_2_ | [M+H]^+^ | 132.1015 | -2.7 | 56.0524, 69.0725, 86.0983 | √ | √ | √ |
| 88 | 4.22 | Uracil | C_4_H_4_N_2_O_2_ | [M+H]^+^ | 113.0343 | -2.2 | 54.0379, 70.0307, 81.0454, 96.0097 | √ | √ | √ |
| 89 | 4.45 | Protocatechuic acid | C_7_H_6_O_4_ | [M-H]^-^ | 153.02 | 4.5 | 67.0220, 109.0310 | √ | ND | √ |
| 90 | 5.64 | Guanosine | C_10_H_13_N_5_O_5_ | [M+H]^+^ | 284.0991 | 0.5 | 110.0350, 135.0300, 152.0564 | √ | √ | √ |
| 91 | 5.92 | Adenine | C5H5N5 | [M+H]+ | 136.0616 | -0.7 | 65.0159, 92.0250, 119.0349 | √ | √ | √ |
| 92 | 6.52 | Nicotinamide | C_6_H_6_N_2_O | [M+H]^+^ | 123.0552 | -1.1 | 78.8068, 80.0512, 106.0281 | √ | √ | √ |
| 93 | 6.72 | Ferulic acid | C_10_H_10_O_4_ | [M-H]^-^ | 193.051 | 1.8 | 76.9729, 134.0373, 178.0264 | √ | √ | √ |
| 94 | 6.88 | Phenylalanine | C_9_H_11_NO_2_ | [M+H]^+^ | 166.0844 | -1.4 | 77.0398, 91.0552, 103.0547, 120.0805 | √ | √ | √ |
|  |  |  |  | [M-H]^-^ | 164.0718 | 0.9 | 147.0460, 103.0571, 91.0568, 72.0119 |  |  |  |
| 95 | 8.08 | Adenine nucleoside | C_10_H_13_N_5_O_4_ | [M-H]^-^ | 266.0894 | -0.4 | (-) | √ | √ | √ |
