# Supplementary figures and images for: Elucidating the phytochemical profile of Sophorae Flavescentis Radix-Astragali Radix herb pair: an integrated LC-QTOF-MS/MS, pharmacological activity, and network pharmacology study on anti-hepatocellular carcinoma effects
Source: Front Chem. 2025 Nov 7;13:1687098. doi: 10.3389/fchem.2025.1687098 (PMC12634525; doi:10.3389/fchem.2025.1687098)

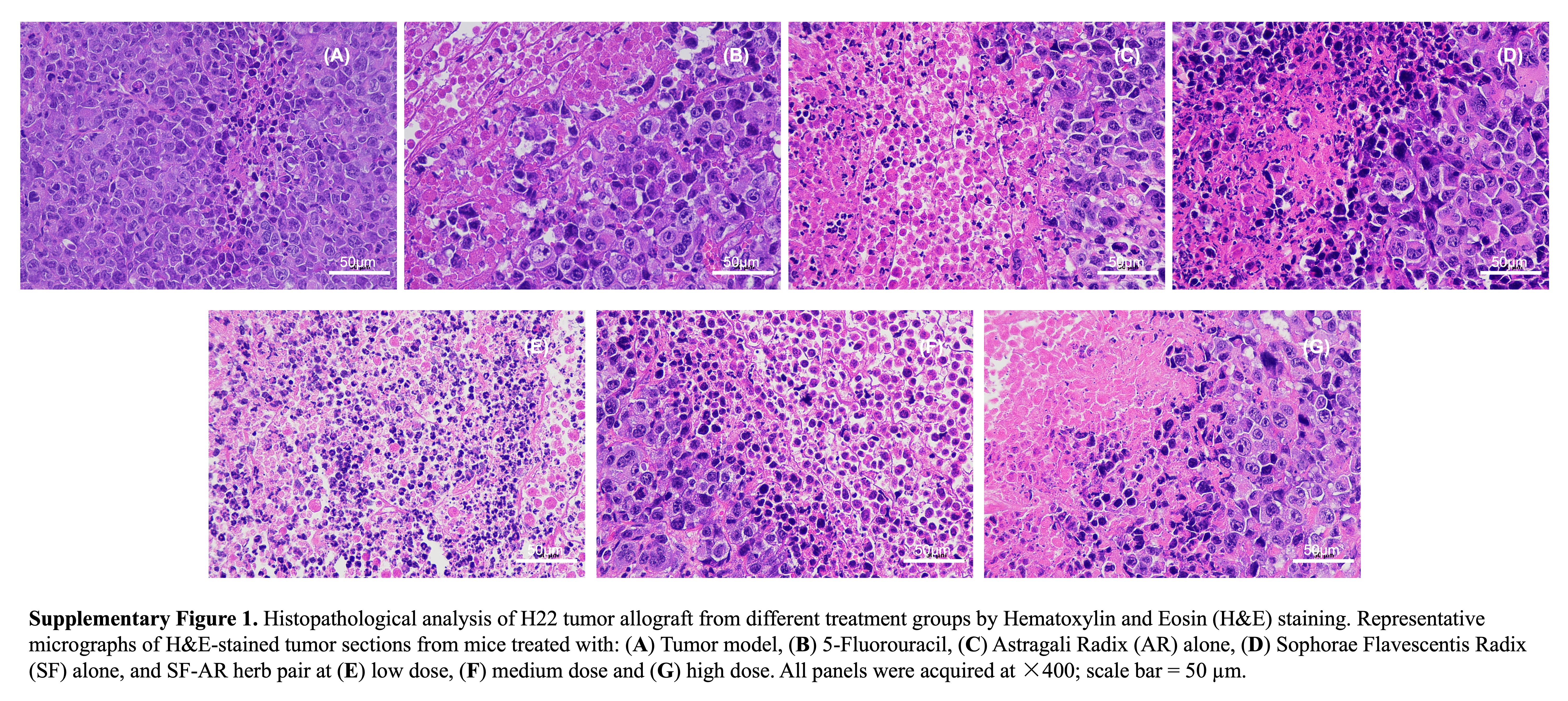

Supplement: Supplementary file 2 [file Image1.jpeg]
