# Supplementary material for: Elucidating the phytochemical profile of Sophorae Flavescentis Radix-Astragali Radix herb pair: an integrated LC-QTOF-MS/MS, pharmacological activity, and network pharmacology study on anti-hepatocellular carcinoma effects
Source: Front Chem. 2025 Nov 7;13:1687098. doi: 10.3389/fchem.2025.1687098 (PMC12634525; doi:10.3389/fchem.2025.1687098)
Supplement: Supplementary file 3 [file DataSheet1.docx]

Supplementary Material

# Chemicals and reagents

HPLC-grade acetonitrile and methanol were obtained from Fisher Scientific (NJ, USA). HPLC-grade ammonium acetate and ammonia solution were purchased from Kemiou Chemical Reagent Co., Ltd. (Tianjin, China). Distilled water was acquired from Wahaha Co., Ltd. (Hangzhou, China). Normal saline (NS) was supplied by Minkang Pharmaceutical Co., Ltd. (Liaoning, China), and 5-fluorouracil (5-FU) was procured from Shanghai Xudong Haipu Pharmaceutical Co., Ltd. (Shanghai, China). Reference standards for constituents anticipated in the SF-AR herb pair are detailed in Supplementary Table S1. The dried, prepared root slices of *Sophora flavescens* Ait. (SF) and *Astragalus membranaceus* (Fisch.) Bge. (AR) were purchased from Guo-Da Drug Store (Shenyang, China). All herbal materials were authenticated and confirmed to comply with the standards stipulated in the Chinese Pharmacopoeia 2020 edition.
